# Explicit Future Pattern-Enhanced Multivariate Time Series Forecasting

**DOI:** 10.3390/e28091031

**Published:** 2026-09-19

**Authors:** Yaokang Li, Yunan Wei, Jing Cai, Wenbin Cheng, Yiting Lin

**Affiliations:** 1 School of Artificial Intelligence, Guangzhou Institute of Science and Technology, Guangzhou 510540, China; yaokangli@gzist.edu.cn (Y.L.); caijing@gzist.edu.cn (J.C.); 2 School of Computer, Zhuhai College of Science and Technology, Zhuhai 519041, China; weiyn@zcst.edu.cn; 3 University of Electronic Science and Technology of China, Zhongshan Institute, Zhongshan 528402, China; 4 University of Electronic Science and Technology of China, Chengdu 611731, China; 5 Guangdong Provincial/Zhuhai Key Laboratory of Interdisciplinary Research and Application for Data Science, Beijing Normal-Hong Kong Baptist University, Zhuhai 519087, China; 6 Department of Computer Science, Hong Kong Baptist University, Hong Kong, China

**Keywords:** multivariate time series, forecasting, future pattern enhanced

## Abstract

Multivariate time series forecasting requires models to infer future values and how the temporal structure evolves beyond the observation boundary. A central challenge is to define this evolution as an intermediate prediction and connect it to value forecasting. We propose explicit future pattern (EFP)-enhanced forecasting, which represents evolution as a multiscale change from the last observed pattern. A separately supervised predictor estimates this change from history, and a decoder combines the predicted pattern with historical behavior and variable relations. A mixed training strategy exposes the decoder to supervised, predicted, and perturbed patterns; inference requires only historical input. Experiments on nine public datasets and four forecasting horizons evaluate the forecasting accuracy and the contribution of explicit future patterns. Predicted patterns outperform shuffled patterns on all nine datasets, and the error increases in aggregate as sample alignment is weakened. Ablations support the roles of dynamic multiscale evolution, frequency information, and mixed training. These results indicate that explicit future patterns can provide useful, inspectable guidance for multivariate forecasting when their estimates remain aligned with the current sample.

## 1. Introduction

Multivariate time series forecasting predicts the future values of several related variables from a shared historical window. It supports planning and decision-making in energy systems, weather services, transportation networks, and economic analysis. Recent reviews and forecasting benchmarks also show that performance depends on both the model and the temporal properties of the data [1,2]. Long-horizon forecasting is especially difficult because trends, periodic behavior, and short variations can occur together. These structures may differ across variables and may continue to change after the last observation. A forecasting model must therefore infer not only future values but also how the underlying temporal pattern evolves beyond the observed interval.

Recent methods address this challenge from several directions. DLinear uses decomposition and linear projection, while PatchTST learns local temporal patterns from patches [3,4]. iTransformer and xCPD strengthen the modeling of relations among variables [5,6]. TimesNet, TimeMixer, and PDF organize temporal information through periodic, multiscale, or frequency-based representations [7,8,9]. Multiscale forecasting has also remained an active topic in recent *Entropy* studies [10]. These designs organize historical signals and combine forecasts at different scales. A complementary question is how to define future pattern evolution as a target of its own and then use its estimate to guide value forecasting.

**Figure 1:**
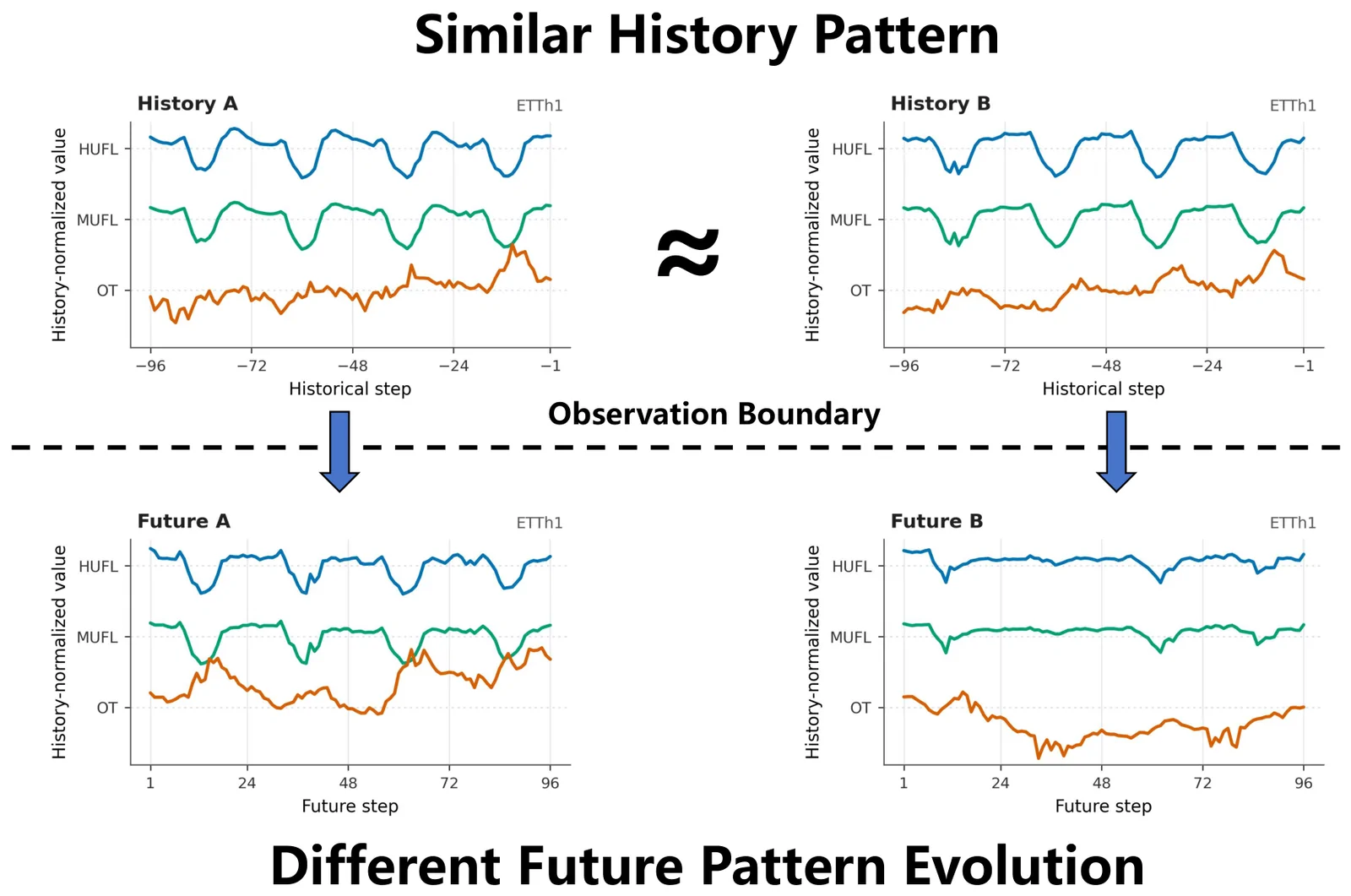
Motivation example using two real ETTh1 samples. Although the samples exhibit similar multivariate patterns over their historical windows, their future patterns evolve differently after the observation boundary. HUFL and MUFL are transformer load variables, and OT is the oil temperature. Each variable is normalized using the mean and standard deviation of its corresponding historical window, and the same statistics are applied to the future interval. All curves are observations from the dataset rather than model outputs.

Figure 1 illustrates this issue with two real ETTh1 samples: their historical multivariate patterns are similar, but their subsequent patterns evolve differently.

Future supervision offers several ways to guide this process. TimeAlign aligns forecasting features with representations learned by reconstructing the future sequence, while L-Drive encodes historical increments into a latent context for forecasting [11,12]. Future-guided learning (FGL) transfers predictions from a teacher with access to later observations to a student forecasting from an earlier window [13]. These approaches motivate a more specific design question: what future representation should be predicted from history and retained as an input to the value decoder? We use the multiscale pattern change relative to the last observed state. This gives each component a defined temporal meaning and supplies a separate supervision signal for its prediction.

The true future pattern is available only when the target sequence is known. At inference, it must be estimated from the historical window. The predicted pattern may therefore differ from the supervised pattern and may lose information that is specific to the current sample. This issue is related to the wider difference between ideal training inputs and model-generated inputs [14]. Changes in time series distributions can make the estimation problem more difficult [15]. A useful forecasting framework must therefore solve two connected problems: it must estimate future pattern evolution from history, and it must train the value predictor to use patterns that contain realistic errors.

To address these problems, we propose EFP, an explicit future pattern-enhanced framework for multivariate time series forecasting. We decompose the historical sequence into multiscale components and repeat the last observed pattern as a persistence reference. A pattern predictor estimates how this reference will change across the forecasting horizon. In parallel, a historical encoder summarizes temporal behavior and variable relations. The forecast decoder combines the historical representation with the predicted future pattern to generate the final values. A mixed training strategy exposes the decoder to supervised, predicted, and perturbed patterns. At inference, all required information is obtained from the historical input.

The main contributions of this work are summarized as follows:

- We define future pattern evolution as the change in fixed multiscale components relative to a persistence reference. This target separates the last observed state from the change to be predicted at each future step.
- We learn this target with a separate pattern predictor and retain its output in the forecast decoder at inference. The decoder combines the predicted evolution with historical features through a learned gate at each future step.
- We train the decoder with supervised, predicted, and perturbed patterns to connect accurate pattern guidance with the errors encountered at inference. Pattern alignment, quality, representation, and training ablations examine how this interface contributes to forecasting.

Experiments on nine public datasets and four forecasting horizons support this formulation. The proposed method (EFP) is evaluated against forecasting models with different architectures, alongside tests of how the predicted patterns guide the final forecast. The aligned predicted pattern outperforms its shuffled counterpart on all nine datasets, while the forecasting error rises in aggregate as pattern alignment is progressively weakened. Further ablations show the value of dynamic multiscale evolution, frequency information, and mixed training. Together, these results show that an explicit estimate of future pattern evolution can provide useful guidance for multivariate forecasting.

## 2. Related Work

### 2.1. Forecasting Methods

Modern time series forecasting methods improve the mapping from historical observations to future values through several model families. DLinear shows that decomposition followed by linear projection can provide a strong and simple baseline, while FITS performs compact interpolation in the frequency domain [3,16]. These works also show that forecasting gains do not always require a large attention model. A suitable representation of the temporal signal can be equally important.

Attention and convolution models provide more flexible ways to construct this representation. PatchTST applies attention to temporal patches within each variable, whereas iTransformer applies attention across tokens representing entire variables [4,5]. Their forecasting heads map the resulting historical features to future values. EFP retains a historical representation too, but adds an independently supervised prediction of future multiscale change to the decoder input. TimesNet maps temporal variation into a two-dimensional space to model several periods, and ModernTCN uses convolutions with large receptive fields [7,17]. These architectural choices concern how observations are encoded. The future pattern path concerns which intermediate quantity is predicted before the final values. Recent reviews also emphasize the relation between temporal representation and forecasting performance [1].

Multivariate forecasting adds the problem of learning how variables interact. Crossformer builds dependencies across both time and variables, while Pathformer selects adaptive paths across temporal scales [18,19]. xCPD routes interactions among channel patches according to their spectral responses, and DUET models temporal distributions together with frequency-based channel relations [6,20]. Intermediate representations have also been studied in *Entropy*—for example, through an information bottleneck objective for compact multivariate features [21]. EFP uses DUET’s channel clustering module to compute the historical dependency mask. Its future pattern target, separate predictor, decoder interface, and mixed training strategy operate alongside this historical encoding step.

### 2.2. Multiscale Modeling

Temporal decomposition separates a sequence into components with different forms of variation. Autoformer places series decomposition inside a forecasting Transformer, and ST-MTM uses seasonal and trend decomposition to guide masked time series representation learning [22,23]. Leddam learns decomposition together with variations within each series and dependencies across series [24]. These methods use decomposition to simplify a complex historical signal before or during representation learning.

Several methods also build the forecast through multiple scales. TimeMixer mixes seasonal and trend features across historical resolutions and combines the outputs of several future predictors [8]. N-HiTS uses input pooling and hierarchical interpolation to assemble forecast components at different scales [25]. FEDformer combines seasonal and trend decomposition with frequency processing inside a Transformer [26]. Thus, predicting through multiscale components is already an established approach. EFP assigns a separate supervised target to the change in each fixed component and supplies the predicted changes to a value decoder together with historical features.

The choice of scale can also be adapted to the signal. AMD learns an adaptive multiscale decomposition, while related studies in *Entropy* combine short- and long-range components or separate trends from faster fluctuations [10,27,28]. EFP uses a fixed decomposition so that the historical reference and the future target share the same component definitions. Its learned gates then adjust how these components contribute to the historical representation and the final forecast.

Frequency modeling offers a complementary description of these structures. FITS performs frequency interpolation with a compact model, while PDF separates temporal variations according to their periods [9,16]. TimeKAN learns frequency components at several levels, FilterTS applies frequency filtering to multivariate signals, and DERITS represents derivatives in the frequency domain for non-stationary series [29,30,31]. FreEformer further combines frequency information with Transformer-based forecasting [32]. Wavelet-based studies in *Entropy* also show that temporal structures can become easier to distinguish after they are separated by scale [33,34].

These approaches establish scale and frequency as useful ways to represent temporal variation. In EFP, they serve two connected roles: the fixed decomposition defines the future change target, and the frequency gate helps to estimate this target from the history. The value decoder receives both the persistence reference and the predicted change. It can therefore learn how to use the estimated evolution together with the historical representation.

### 2.3. Future Pattern Modeling

Future supervision can shape either the training objective or an intermediate prediction. FGL uses a teacher operating at a later observation time to guide a student forecasting the same target from an earlier time. Teacher predictions provide soft supervision through a distillation loss, and the student performs forecasting at deployment [13]. EFP instead defines its supervised target directly through the fixed decomposition of the training sequence. The pattern predictor estimates this target from the history, and its output remains an explicit decoder input at deployment.

TimeAlign uses a future reconstruction branch to produce features that guide the historical forecasting branch through global and local alignment losses. The reconstruction branch is used during training, while the forecasting branch remains at inference [11]. EFP learns a numerical change for every future step and scale, rather than aligning learned embeddings. It first trains this pattern predictor and then freezes it while the historical encoder and decoder learn to use its estimates. This gives the intermediate output a stable meaning throughout decoder training.

L-Drive constructs a latent context from normalized historical observations and their increments. Gated increments are processed by a GRU, and the resulting context is combined with historical values for patch-based forecasting [12]. Both L-Drive and EFP use a representation of change to guide prediction. Their change representations differ in temporal position and supervision: L-Drive encodes observed increments into historical context, whereas EFP predicts the future changes in fixed multiscale components against a target derived from the training future.

Transforming the future target can also improve the loss used to train a forecaster. Time-o1 aligns transformed predictions and labels along selected decorrelated components [35]. Such objectives supervise the final forecast in an additional representation space. In a conventional multitask design, an auxiliary target can likewise shape a shared encoder without becoming an input to the main prediction head. EFP uses a serial connection: the independently trained pattern prediction is consumed by the value decoder. This connection makes the accuracy and sample alignment of the intermediate prediction directly relevant to value forecasting.

A related body of work studies changes between historical and future distributions. RevIN normalizes each sample with reversible statistics, Dish-TS learns to reduce the distribution gap between historical and future windows, and Non-Stationary Transformer combines normalization with the recovery of non-stationary dependencies [36,37,38]. TimeBridge further emphasizes the effects of non-stationarity on long-horizon forecasting [15]. These methods address changes in values, statistics, or latent distributions. Future pattern modeling asks a connected question at a different level: how should the structural change after the boundary be represented, estimated, and used by the value predictor?

The pattern used to guide training can also be more accurate than the estimate available at inference. A similar difference between ideal training inputs and model-generated inputs appears in sequence prediction. Scheduled sampling reduces this difference by exposing a recurrent model to its own outputs during training [14]. In our setting, the generated input is not a previous output token. It is a full estimate of future pattern evolution over the forecast horizon. The decoder must learn both the meaning of an accurate pattern and the errors produced by the pattern predictor.

These distinctions place EFP at the connection between intermediate prediction and value forecasting. A fixed multiscale target defines what to predict; a separate predictor estimates it from history; and mixed training teaches the decoder to use estimates with realistic errors. The experiments examine this connection through pattern alignment, quality, representation, and the training strategy.

## 3. Problem Formulation

This section formalizes multivariate forecasting using an explicit estimate of how multiscale patterns evolve after the observation boundary. We first define the forecasting task and its deployment setting; we then represent future pattern evolution relative to persistence and describe three aspects that govern its use in forecasting: pattern alignment, pattern quality, and mixed pattern training.

### 3.1. Time Series Forecasting

A time series is an ordered sequence of observations over time. We write it as

(1) $$Z={\left[z_{1},\ldots ,z_{T}\right]}^{⊤}\in R^{T\times N},\;z_{\tau }\in R^{N}.$$

Here, *T* is the number of timestamps and *N* is the number of channels. The first index denotes the time and the second index denotes the channel. Thus, $Z_{\tau ,n}$ is the value of channel *n* at timestamp τ, $Z_{:,n}\in R^{T}$ is the full sequence of channel *n*, and $Z_{\tau ,:}\in R^{N}$ is the observation at timestamp τ. A time series is univariate when N=1 and multivariate when N>1.

For a forecasting sample, we divide a multivariate time series into a historical window and its following future window:

(2) $$\begin{array}{cc} X & ={\left[z_{1},\ldots ,z_{L}\right]}^{⊤}\in R^{L\times N},\\ Y & ={\left[z_{L+1},\ldots ,z_{L+H}\right]}^{⊤}\in R^{H\times N}. \end{array}$$

Here, *L* is the lookback length and *H* is the forecasting horizon. Multivariate time series forecasting aims to predict Y from X. A standard forecasting model learns the mapping

(3) $$f_{\Theta }:R^{L\times N}\to R^{H\times N},\;\hat{Y}=f_{\Theta }\left(X\right),$$

using a value loss such as the MSE or MAE.

### 3.2. Pattern Decomposition

We represent temporal patterns using a fixed Haar/db1 decomposition W with left replication and without downsampling. For a sequence $S\in R^{T\times N}$, let $A_{0}=S$ and let $d_{j}=2^{j-1}$ be the dilation at level *j*. We define the extension on the left by ${\bar{A}}_{j-1}\left(q,n\right)=A_{j-1}\left(1,n\right)$ for q<1 and ${\bar{A}}_{j-1}\left(q,n\right)=A_{j-1}\left(q,n\right)$ otherwise. The approximation and detail components are

(4) $$\begin{array}{cc} A_{j}\left(q,n\right)\; & =\frac{{\bar{A}}_{j-1}\left(q-d_{j},n\right)+A_{j-1}\left(q,n\right)}{\sqrt{2}}, \end{array}$$

(5) $$\begin{array}{cc} D_{j}\left(q,n\right)\; & =\frac{{\bar{A}}_{j-1}\left(q-d_{j},n\right)-A_{j-1}\left(q,n\right)}{\sqrt{2}}, \end{array}$$

for q∈{1,…,T}, n∈{1,…,N}, and j∈{1,…,J}. The approximation component applies the low-pass filter $\left[1,1\right]/\sqrt{2}$, whereas the detail component applies the high-pass filter $\left[1,-1\right]/\sqrt{2}$. The second expression follows the implementation and computes the past value minus the current value. The transform preserves the temporal length and returns

(6) $$W\left(S\right)=\left[D_{1},\ldots ,D_{J},A_{J}\right]\in R^{T\times N\times K},\;K=J+1.$$

We use four levels (J=4), giving five components (K=5), $D_{1}$, $D_{2}$, $D_{3}$, $D_{4}$, and $A_{4}$, in sequence.

Applying the decomposition to the historical input gives

(7) $$C_{X}=W\left(X\right)\in R^{L\times N\times K}.$$

We use the final observed multiscale state as a persistence reference and repeat it across the forecasting horizon:

(8) $$C_{\mathrm{pers}}\left[h,:,:\right]=C_{X}\left[L,:,:\right],\;h=1,\ldots ,H,$$

where $C_{\mathrm{pers}}\in R^{H\times N\times K}$. This reference separates the multiscale state already observed at the forecast origin from the structural change that must be estimated beyond it.

### 3.3. Future Pattern

For a paired training example, we concatenate the historical and future windows and apply the same decomposition:

(9) $$S=\left[X;Y\right]\in R^{(L+H)\times N},\;C_{Y}^{★}={\left[W(S)\right]}_{L+1:L+H,:,:}\in R^{H\times N\times K}.$$

Because the transform uses the left context, this construction preserves the observed samples immediately preceding the forecasting boundary when computing the first future components. The supervised future pattern change is defined relative to persistence:

(10) $$\Delta^{★}=C_{Y}^{★}-C_{\mathrm{pers}}\in R^{H\times N\times K}.$$

A future pattern predictor $p_{\phi }$ estimates this change from historical components,

(11) $$\hat{\Delta }=p_{\phi }\left(C_{X}\right),\;\hat{\Delta }\in R^{H\times N\times K},$$

and forms the deployable future pattern representation

(12) $$C_{\mathrm{pred}}=\left[C_{\mathrm{pers}}∥\hat{\Delta }\right]\in R^{H\times N\times 2K},$$

where || denotes concatenation along the component dimension. The final forecast combines a historical representation $e_{\psi }\left(X\right)$ with this representation:

(13) $$\hat{Y}=d_{\theta }\left(e_{\psi }\left(X\right),C_{\mathrm{pred}}\right).$$

During training, $\Delta^{★}$ supplies direct pattern supervision and defines the oracle future pattern representation $C_{\mathrm{oracle}}=\left[C_{\mathrm{pers}}∥\Delta^{★}\right]$ for interface learning and diagnostic evaluation. During inference, the same interface is driven by $C_{\mathrm{pred}}$. For fixed predictor parameters, this representation is a deterministic function of X. Future supervision shapes the learned mapping, while the deployed pattern path organizes the historical signal into an estimate of future change. This structured intermediate prediction is the inductive bias introduced by EFP.

This formulation highlights three aspects of explicit future pattern modeling.

1. Pattern alignment. A predicted pattern change is associated with the historical sample from which it is estimated. Shuffling estimates across samples breaks this correspondence and removes future guidance specific to the sample.
2. Pattern quality. Let $\Pi (\hat{\Delta })$ denote an estimate shuffled within the batch and define
  (14) $${\hat{\Delta }}^{\left(\rho \right)}=\left(1-\rho \right)\hat{\Delta }+\rho \Pi \left(\hat{\Delta }\right),\;\rho \in \left[0,1\right].$$
  This interpolation moves continuously from the original prediction at ρ=0 to a shuffled estimate at ρ=1, providing a controlled view of how the forecasting performance changes with pattern quality.
3. Mixed pattern training. We train the forecasting model with oracle, predicted, and perturbed future patterns. Their combination exposes the forecaster to both accurate patterns and realistic estimation errors, helping it to use the predicted patterns at inference.

Together, these aspects connect the representation of future pattern evolution with its use in forecasting. Section 4 describes how future patterns are estimated and incorporated, while Section 5 examines pattern alignment, pattern quality, and the mixed training strategy.

## 4. Method

### 4.1. Overview

Our method (EFP) makes future pattern evolution an explicit input to value forecasting. Given $X\in R^{B\times L\times N}$, where *B* denotes the batch size, the model follows two coordinated paths:

(15) $$\;\left.\begin{array}{cc} X & \overset{\;e_{\psi }\;}{\to }Z_{\mathrm{hist}},\\ X & \overset{\;W\;}{\to }C_{X}\overset{\;{\mathrm{Repeat}}_{H},\;p_{\phi }\;}{\to }\left(C_{\mathrm{pers}},\hat{\Delta }\right)\overset{\;\mathrm{Concat}\;}{\to }C_{\mathrm{pred}} \end{array}\right\}\overset{\;d_{\theta }\;}{\to }\hat{Y}.$$

The historical path maps X to the variable representations $Z_{\mathrm{hist}}$. In parallel, the future pattern path maps the same input to $C_{\mathrm{pred}}$, which describes the multiscale state expected over the forecasting horizon. The forecast decoder combines these two outputs and produces $\hat{Y}$. The parameters of $p_{\phi }$ are shared across variables, so every variable is processed by the same pattern predictor.

**Figure 2:**
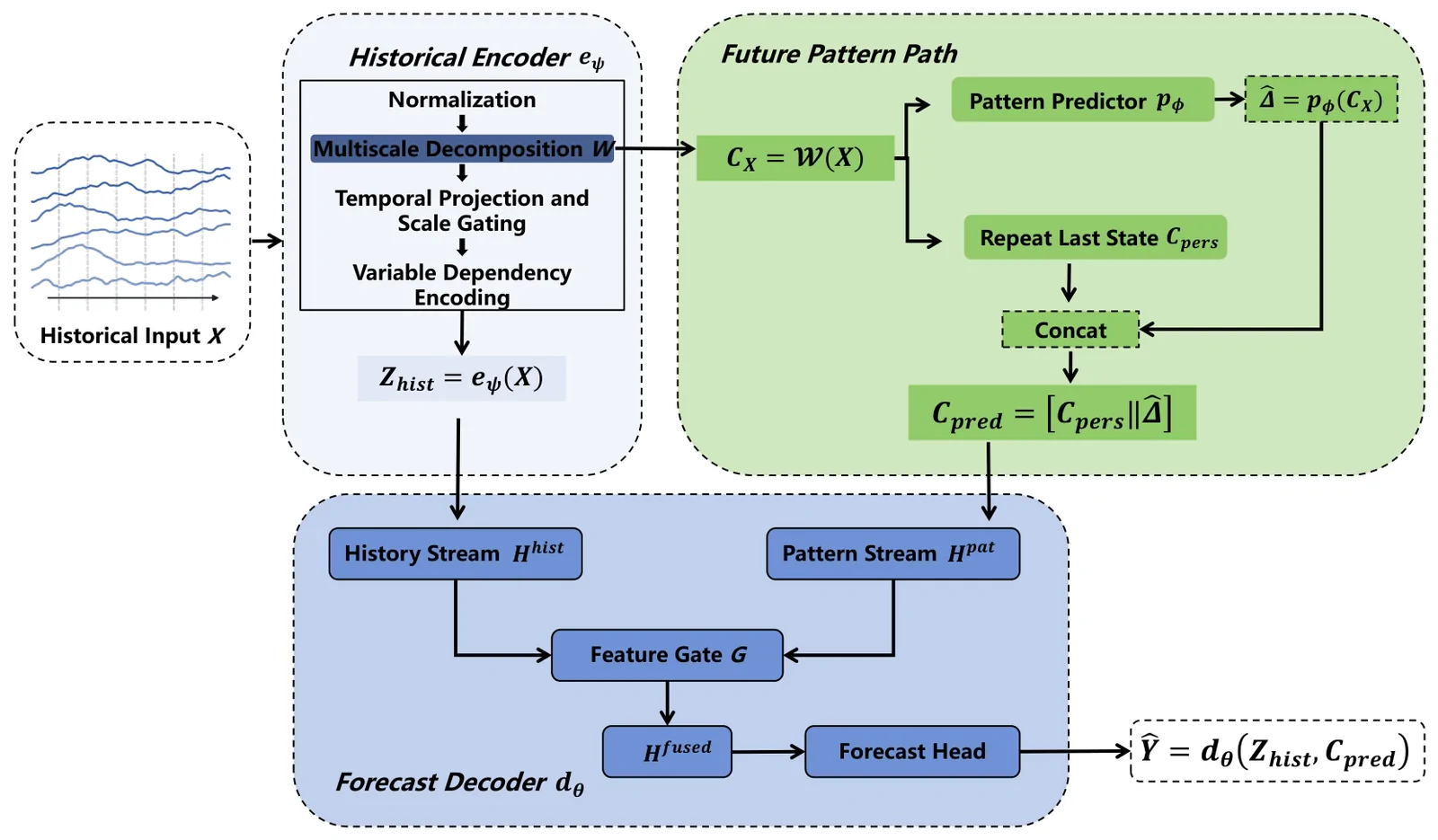
Overall architecture of the proposed framework. The historical encoder extracts multiscale temporal and variable dependency representations, while the future pattern path estimates multiscale changes relative to the persistent state. The forecast decoder aligns and fuses the two representations to generate the final prediction.

The complete architecture and data flow are summarized in Figure 2. Training proceeds in two stages. The first stage learns the pattern predictor from the supervised future pattern change. The second stage freezes this predictor and trains the historical encoder and decoder with oracle, predicted, and perturbed future patterns. At inference, the model receives only X. The following subsections follow the architecture from the historical encoder to the future pattern path and then to the forecast decoder. Section 4.5 and Algorithm 1 describe the training and inference procedure.
**Algorithm 1** EFP training and inference**Input:** Training and validation windows, lookback *L*, horizon *H*, and decomposition W.**Output:** Pattern predictor $p_{\phi }$, historical encoder $e_{\psi }$, and decoder $d_{\theta }$.For each training batch (X,Y), compute $C_{X}$, repeat its last state to form $C_{\mathrm{pers}}$, and construct $\Delta^{★}$ using Equation (23).In Stage 1, predict $\hat{\Delta }=p_{\phi }\left(C_{X}\right)$ for each batch and update only ϕ using $L_{\mathrm{pat}}$ in Equation (33). Evaluate the pattern RMSE on the validation set after each epoch and apply early stopping.Restore the pattern checkpoint with the lowest validation RMSE. Freeze ϕ and keep $p_{\phi }$ in evaluation mode throughout Stage 2.At each Stage 2 epoch, assign oracle, predicted, and perturbed patterns to batches in a shuffled schedule with proportions 0.25, 0.50, and 0.25.For each batch, form $\tilde{\Delta }$ using Equation (34). For perturbed patterns, obtain $E_{r}$ from an independently sampled training batch and draw *s* uniformly from {0.5,1.0}. Construct $\tilde{C}=\left[C_{\mathrm{pers}}∥\tilde{\Delta }\right]$, compute $\hat{Y}=d_{\theta }\left(e_{\psi }\left(X\right),\tilde{C}\right)$, and update only (ψ,θ) using $L_{\mathrm{forecast}}$.After each Stage 2 epoch, evaluate the validation MSE with predicted patterns and apply early stopping. Restore the encoder and decoder checkpoint with the lowest such MSE.At inference, compute $C_{X}=W\left(X\right)$ and repeat its last state to form $C_{\mathrm{pers}}$. Estimate $\hat{\Delta }=p_{\phi }\left(C_{X}\right)$, form $C_{\mathrm{pred}}=\left[C_{\mathrm{pers}}∥\hat{\Delta }\right]$, and return $d_{\theta }\left(e_{\psi }\left(X\right),C_{\mathrm{pred}}\right)$.

### 4.2. Historical Encoder

The historical encoder converts the lookback window into one representation of dimension *d* for each variable. As shown in Figure 2, it contains four steps: normalization, multiscale decomposition, projection and gating, and variable dependency encoding. The first three steps summarize the temporal pattern of each variable. The last step models interactions among variables.

**Normalization.** Although the input has already been standardized at the dataset level, different lookback windows may still have different local statistics. We therefore normalize each variable within its own lookback window:

(16) $$\tilde{X}=\mathrm{Norm}\left(X\right)\in R^{B\times L\times N}.$$

The operator Norm(·) removes the temporal mean and rescales the temporal variance of each sample and variable. Gradients are not propagated through the window statistics. A learned scale and bias are then applied to each variable. This local operation follows the dataset-level standardization described in Section 5.1.

**Multiscale decomposition.** We next apply the fixed decomposition W to the normalized history:

(17) $$\tilde{C}=W\left(\tilde{X}\right)\in R^{B\times L\times N\times K}.$$

The tensor $\tilde{C}$ contains a length *L* sequence for every variable and every multiscale component. In the following notation, *b*, *n*, and *k* index a sample, a variable, and a component, respectively.

**Projection and gating.** Each component is first projected along the temporal dimension:

(18) $$P_{bnk}={\mathrm{Proj}}_{k}\left({\tilde{C}}_{b,:,n,k}\right)\in R^{d}.$$

The projection ${\mathrm{Proj}}_{k}$ is specific to component *k* but shared across samples and variables. We then summarize each component by its temporal mean, standard deviation, change from the first to the last position, and mean absolute value. Concatenating these summaries gives $s_{bn}\in R^{4K}$. A small network converts $s_{bn}$ into the scale weights $a_{bn}\in R^{K}$, which are used to combine the projected components:

(19) $$\begin{array}{cc} a_{bn} & =\mathrm{softmax}\left(g_{\mathrm{scale}}\left(s_{bn}\right)\right),\\ T_{bn} & =\sum_{k=1}^{K}a_{bnk}P_{bnk}\in R^{d}. \end{array}$$

The resulting token $T_{bn}$ summarizes the historical pattern of variable *n*. Because $a_{bn}$ depends on the current sample and variable, the encoder can emphasize different scales for different inputs.

**Variable dependency encoding.** For multivariate inputs, the tokens in $T\in R^{B\times N\times d}$ interact across related variables. The dependency mask follows the channel clustering module of DUET [20]. It compares the historical spectra of variable pairs, normalizes the resulting similarities, and applies hard Gumbel sampling to form $M_{X}\in {\left\{0,1\right\}}^{B\times N\times N}$. Appendix B and Appendix C specify the mask computation and evaluation seed. Masked variable attention then updates each token using the selected variables:

(20) $$Z_{\mathrm{hist}}=\mathrm{DepEnc}\left(T;M_{X}\right)=e_{\psi }\left(X\right)\in R^{B\times N\times d}.$$

The output $Z_{\mathrm{hist}}$ is the historical representation supplied to the forecast decoder. For a univariate input, dependency encoding is unnecessary, and T is returned directly.

### 4.3. Future Pattern Path

The future pattern path converts the historical input into a pattern representation for the forecasting horizon. It follows the lower path in Figure 2: multiscale decomposition, pattern prediction, and concatenation.

**Historical pattern state.** The path starts with applying W to the standardized historical input:

(21) $$C_{X}=W\left(X\right)\in R^{B\times L\times N\times K}.$$

The tensor $C_{X}$ records the *K* multiscale components at every historical timestamp. Its last temporal state describes the pattern observed at the forecast origin. We repeat this state across the *H* future positions to form the persistence reference:

(22) $$C_{\mathrm{pers}}\left[:,h,:,:\right]=C_{X}\left[:,L,:,:\right],\;h=1,\ldots ,H.$$

Here, *h* indexes a future position. This reference represents the simple case in which the last observed pattern remains unchanged over the forecasting horizon.

**Pattern prediction.** The pattern predictor $p_{\phi }$ estimates how the future pattern departs from $C_{\mathrm{pers}}$. During training, the target change is constructed from the historical input X and the future target Y:

(23) $$C_{Y}^{★}={\left[W\left(\left[X;Y\right]\right)\right]}_{:,L+1:L+H,:,:},\;\Delta^{★}=C_{Y}^{★}-C_{\mathrm{pers}}.$$

Here, [X;Y] denotes temporal concatenation. The tensor $C_{Y}^{★}\in R^{B\times H\times N\times K}$ contains the multiscale components over the future interval. Subtracting $C_{\mathrm{pers}}$ gives the supervised pattern change $\Delta^{★}$. The future target is used only during training. It supervises $p_{\phi }$ in the first stage and is used to construct the oracle and perturbed pattern sources in the second stage. At inference, $\hat{\Delta }$ is estimated entirely from X.

To estimate this change from history, $p_{\phi }$ first expresses every state in $C_{X}$ relative to its last observed state:

(24) $$C^{c}\left[:,q,:,:\right]=C_{X}\left[:,q,:,:\right]-C_{X}\left[:,L,:,:\right],\;q=1,\ldots ,L.$$

Here, *q* indexes a historical position. The centered sequence $C^{c}$ shows how each component approached its state at the forecast origin. The predictor also constructs a frequency view $C^{f}$ and a difference view $C^{d}$:

(25) $$\begin{array}{cc} C^{f} & =\mathrm{irFFT}\left(\mathrm{rFFT}\left(C^{c}\right)⊙\sigma \left(\Theta_{f}\right)\right),\\ C^{d} & =\nabla_{t}C^{c}. \end{array}$$

The Fourier transforms operate along the temporal dimension. The function σ(·) is the sigmoid function, and ⊙ denotes elementwise multiplication. The learned gate $\sigma (\Theta_{f})$ controls the contribution of each frequency and component. The operator $\nabla_{t}$ is the first temporal difference, with the first position set to zero. The three views are concatenated as $V=\left[C^{c}∥C^{f}∥C^{d}\right]\in R^{B\times L\times N\times 3K}$.

The predictor uses a linear branch and a nonlinear branch. The linear branch maps $C^{c}$ directly from the *L* historical positions to the *H* future positions and produces $\Delta^{\mathrm{lin}}$. The nonlinear branch processes V with temporal convolution blocks and produces a correction R. Both tensors have shape B×H×N×K. Their outputs are combined by a learned component gate:

(26) $$\hat{\Delta }=p_{\phi }\left(C_{X}\right)=\Delta^{\mathrm{lin}}+\sigma \left(\eta \right)⊙R\in R^{B\times H\times N\times K},$$

where $\eta \in R^{K}$. During training, $\Delta^{★}$ supervises $\hat{\Delta }$. At inference, $p_{\phi }$ obtains $\hat{\Delta }$ entirely from $C_{X}$.

**Future pattern representation.** The final step joins the repeated state and its predicted change along the component dimension:

(27) $$C_{\mathrm{pred}}=\mathrm{Concat}\left(C_{\mathrm{pers}},\hat{\Delta }\right)=\left[C_{\mathrm{pers}}∥\hat{\Delta }\right]\in R^{B\times H\times N\times 2K}.$$

Thus, $C_{\mathrm{pred}}$ carries two complementary pieces of information at every future position: the last observed multiscale state and its estimated change. This representation is passed to the pattern stream of the forecast decoder.

### 4.4. Forecast Decoder

The forecast decoder receives $Z_{\mathrm{hist}}$ from the historical encoder and $C_{\mathrm{pred}}$ from the future pattern path. It first maps them into two representations with the same shape: the history stream $H_{\mathrm{hist}}$ and the pattern stream $H_{\mathrm{pat}}$. Both are defined for every future position and variable.

**History stream.** We project each historical variable token, repeat it across the forecasting horizon, and add a learned horizon embedding $E\in R^{H\times d}$:

(28) $$H_{\mathrm{hist}}={\mathrm{Repeat}}_{H}\left({\mathrm{Proj}}_{\mathrm{hist}}\left(Z_{\mathrm{hist}}\right)\right)+E\in R^{B\times H\times N\times d}.$$

The horizon embedding distinguishes future positions that share the same historical token.

**Pattern stream.** The pattern stream maps the 2K features in $C_{\mathrm{pred}}$ to dimension *d*. It then applies temporal convolution along the forecasting horizon to connect neighboring future positions:

(29) $$H_{\mathrm{pat}}=E_{\mathrm{pat}}\left(C_{\mathrm{pred}}\right)\in R^{B\times H\times N\times d}.$$

The encoder $E_{\mathrm{pat}}$ uses a residual connection and normalization after temporal processing. At this point, $H_{\mathrm{hist}}$ and $H_{\mathrm{pat}}$ are aligned by sample, future position, variable, and hidden feature.

**Feature fusion.** A learned gate is computed from the two aligned streams:

(30) $$G=\sigma \left(g_{\mathrm{fuse}}\left(\left[H_{\mathrm{hist}}∥H_{\mathrm{pat}}\right]\right)\right)\in R^{B\times H\times N\times d}.$$

The gate is computed separately for every sample, future position, variable, and hidden feature. It produces the fused representation

(31) $$H_{\mathrm{fused}}=\left(1-G\right)⊙H_{\mathrm{hist}}+G⊙H_{\mathrm{pat}}\in R^{B\times H\times N\times d}.$$

**Forecast head.** Finally, a shared forecast head maps every fused feature vector to one value:

(32) $$\hat{Y}=\mathrm{Head}\left(H_{\mathrm{fused}}\right)\in R^{B\times H\times N}.$$

The decoder therefore follows the same order as the architecture in Figure 2: construct the two streams, compute their feature gate, fuse them, and produce $\hat{Y}$.

### 4.5. Mixed Training Strategy

Training provides the supervised change $\Delta^{★}$, whereas inference relies on the estimated change $\hat{\Delta }$. We use two training stages to learn the pattern predictor first and then prepare the forecasting model for patterns with different levels of accuracy. Algorithm 1 summarizes the updates and connects them to the two paths in Figure 2.

In the first stage, we optimize $p_{\phi }$ while keeping the historical encoder and forecast decoder fixed. The predictor minimizes

(33) $$L_{\mathrm{pat}}=SmoothL1\left(\hat{\Delta },\Delta^{★}\right).$$

The checkpoint with the lowest validation RMSE for the pattern change is retained.

In the second stage, we freeze $p_{\phi }$ and optimize the historical encoder $e_{\psi }$ and forecast decoder $d_{\theta }$. Each batch uses an oracle, predicted, or perturbed pattern change. For the perturbed source, an additional training batch $\left(X_{r},Y_{r}\right)$ is sampled independently. Its prediction residual is $E_{r}=p_{\phi }\left(W\left(X_{r}\right)\right)-\Delta_{r}^{★}$. The pattern change supplied to the decoder is

(34) $$\tilde{\Delta }=\left\{\begin{array}{cc} \Delta^{★},\; & \mathrm{oracle},\\ \hat{\Delta },\; & \mathrm{predicted},\\ \Delta^{★}+sE_{r},\; & \mathrm{perturbed}, \end{array}\right.\;s\in \left\{0.5,1.0\right\}.$$

Adding $E_{r}$ to the current oracle change exposes the decoder to errors produced by the fixed predictor. We draw *s* uniformly from {0.5,1.0} once per perturbed batch. Within each epoch, a shuffled batch schedule assigns the oracle, predicted, and perturbed sources in proportions of 0.25, 0.50, and 0.25, rounded to whole batches.

The persistence reference is unchanged for all three sources. We construct the decoder input as $\tilde{C}=\left[C_{\mathrm{pers}}∥\tilde{\Delta }\right]$ and minimize the value forecasting loss

(35) $$L_{\mathrm{forecast}}=\mathrm{MSE}\left(\hat{Y},Y\right).$$

The final checkpoint is selected via the validation MSE using the predicted pattern, which matches the inference setting. Given only X, the trained model computes $\hat{\Delta }$, forms $C_{\mathrm{pred}}$, and produces $\hat{Y}$.

## 5. Experiments

### 5.1. Experimental Settings

We evaluate the proposed method on nine public multivariate time series datasets from the Time Series Forecasting Benchmark (TFB) [2]. These datasets cover energy, finance, meteorology, and transportation, with substantial differences in data scale, sampling frequency, and the number of variables.

The ETT collection contains transformer temperature and power load measurements from two regions in China between 2016 and 2018. It includes two hourly datasets, ETTh1 and ETTh2, and two datasets sampled every 15 min, ETTm1 and ETTm2. Exchange contains 7588 daily observations of exchange rates from eight economies. Weather contains 21 meteorological indicators recorded every 10 min in Germany throughout 2020.

Electricity contains hourly electricity consumption records for 321 clients from 2012 to 2014. Traffic reports hourly road occupancy rates measured by 862 sensors on freeways in the San Francisco Bay Area from 2015 to 2016. Solar-Energy records solar power production from 137 photovoltaic plants in Alabama at 10 min intervals during 2006. Table 1 summarizes the statistical information for all datasets.

We follow the TFB protocol for data partitioning, rolling window construction, and dataset-level standardization. The same processing is used for every compared method. All nine datasets are evaluated at forecasting horizons H∈{96,192,336,720}, giving 36 dataset and horizon settings in the main comparison.

#### 5.1.1. Baselines

We compare our method with six representative forecasting models from several architecture families. The Transformer baselines include iTransformer [5], PatchTST [4], and Crossformer [18]. We also include the convolution-based TimesNet [7], the multiscale mixing model TimeMixer [8], and the decomposition-based linear model DLinear [3]. Together, these methods cover channel modeling, patch modeling, temporal convolution, multiscale mixing, and linear forecasting. We also compare EFP with AMD, FITS, Pathformer, DUET, ModernTCN, TimeBridge, SimpleTM [39], and MOIRAI [40]. These additional comparisons are reported in Table A4 in Appendix D.

#### 5.1.2. Implementation Details

We use the mean squared error (MSE) and mean absolute error (MAE) as the evaluation metrics, where lower values indicate better forecasts. Both metrics are computed in the standardized data space defined by the TFB protocol. Metrics in the original data scale are retained only as implementation diagnostics and are not included in the main comparison.

We rerun all six baselines and search L∈{336,512,720} for each method and dataset. Each candidate is scored by its mean validation MSE over the four horizons, H∈{96,192,336,720}. The candidate with the lowest mean is selected, and its value of *L* is shared across the four horizons and three repeated runs. Model weights are trained separately for each horizon and run. We evaluate the selected configurations on the test set without using test scores for selection. Table A1 in Appendix A lists the selected lookback lengths.

Every experiment is repeated with seeds 2024, 2025, and 2026, and the main tables report the mean result. Each EFP training stage is limited to 300 epochs with early stopping patience of 50 epochs. Both stages use AdamW with a learning rate of $10^{-3}$, weight decay of $10^{-4}$, a batch size of 32, and gradient norm clipping at 1.0. Stage 1 selects the checkpoint via the validation pattern RMSE; Stage 2 uses the validation forecast MSE with the predicted patterns, as detailed in Algorithm 1.

EFP uses a hidden dimension of 64, one variable attention layer with four heads, and dropout of 0.1. The pattern predictor has two temporal convolution blocks with kernel size 5 and dilations 1 and 2. Table A2 and Table A3 in Appendix B and Appendix C give the architecture and training settings, including the dependency mask and random sampling. All experiments use PyTorch 2.11.0 on a single NVIDIA GeForce RTX 4090 GPU.

### 5.2. Main Results

Table 2 reports the forecasting results over the 36 dataset and horizon settings. Each dataset block contains the four prediction horizons and their averages. The final two rows count the first-place MSE and MAE results obtained by every method over the 36 horizon-specific comparisons.

Within the comparison in Table 2, EFP achieves the lowest average ranks. Its average rank is 1.333 for the MSE and 1.208 for the MAE, compared with 2.750 and 3.014 for the second-ranked method, PatchTST. It records 29 first-place MSE results when ties are included and 30 first-place MAE results. Relative to the strongest of these six baselines in each setting, the method obtains 26 wins, 3 ties, and 7 losses in the MSE, together with 30 wins and 6 losses in the MAE.

The aggregate errors in Table 2 give a similar comparison. Our method obtains macro averages of 0.2896 in the MSE and 0.3211 in the MAE, whereas PatchTST, the strongest baseline in terms of the macro average, obtains 0.2988 and 0.3334. These results correspond to reductions of 3.07% and 3.69%, respectively. Within this table, EFP achieves the best or tied best average MSE and the best average MAE on all nine datasets. The consistency across energy, economic, weather, solar, and traffic data shows that the method remains competitive under different temporal structures and forecasting lengths.

Appendix E Table A5 and Appendix F Table A6 report the mean ± SD across three runs for every dataset and horizon in the main and additional comparisons. These results show the variation across runs alongside the average forecasting error.

We also compare EFP with the 14 baselines and the Value Forecast control using paired Wilcoxon tests. The four horizons are averaged within each dataset, and the related ETT datasets are combined into one group. The resulting six benchmark groups are tested with exact two-sided *p* values and Holm correction across all 30 method–metric comparisons. No comparison reaches the corrected 0.05 significance threshold (Appendix G Table A7). The error differences and rankings thus summarize the observed benchmark performance. The following experiments examine how future pattern alignment and quality contribute to this performance.

### 5.3. Future Pattern Quality

We first evaluate how accurately the pattern predictor estimates future changes from history. Table 3 compares $\hat{\Delta }$ with the supervised target $\Delta^{★}$ using the MSE and MAE. Persistence predicts zero change and therefore retains the last observed multiscale state. Both estimates are evaluated on the same test windows in the pattern space obtained from standardized observations. For each run and forecasting horizon, errors are averaged over test windows, future positions, variables, and all five components. We then average these metrics equally over the four horizons and three runs.

The predictor reduces both pattern errors on all nine datasets. The macro average MSE decreases from 0.1806 to 0.1406, and the MAE decreases from 0.2588 to 0.2205, corresponding to reductions of 22.16% and 14.81%. The gains vary across datasets: the MSE decreases by 30.85% on ETTh2 and 4.68% on Electricity. These results show that the estimated changes bring the future multiscale state closer to its observed value than persistence does. They support the learning of pattern evolution from history as an intermediate forecasting task.

We next examine how the decoder uses the estimated future pattern. We vary the source and integrity of the pattern supplied to the same forecasting model while keeping the historical input and persistence state unchanged. Oracle uses the multiscale pattern change obtained from the true future sequence and serves as a diagnostic reference. Predicted uses the change estimated from history and corresponds to the inference setting. Shuffled permutes the predicted changes across samples, preserving the collection of predicted patterns within a batch while breaking their correspondence with the historical samples.

We further construct three intermediate patterns using Equation (14). C25, C50, and C75 mix the predicted change with 25%, 50%, and 75% of the shuffled change, respectively. The replacement acts only on the pattern change, while the persistence state of each sample remains fixed. This controlled sequence separates the value of sample alignment from the generic presence of an additional pattern representation. Table 4 reports the resulting forecasting errors.

**Figure 3:**
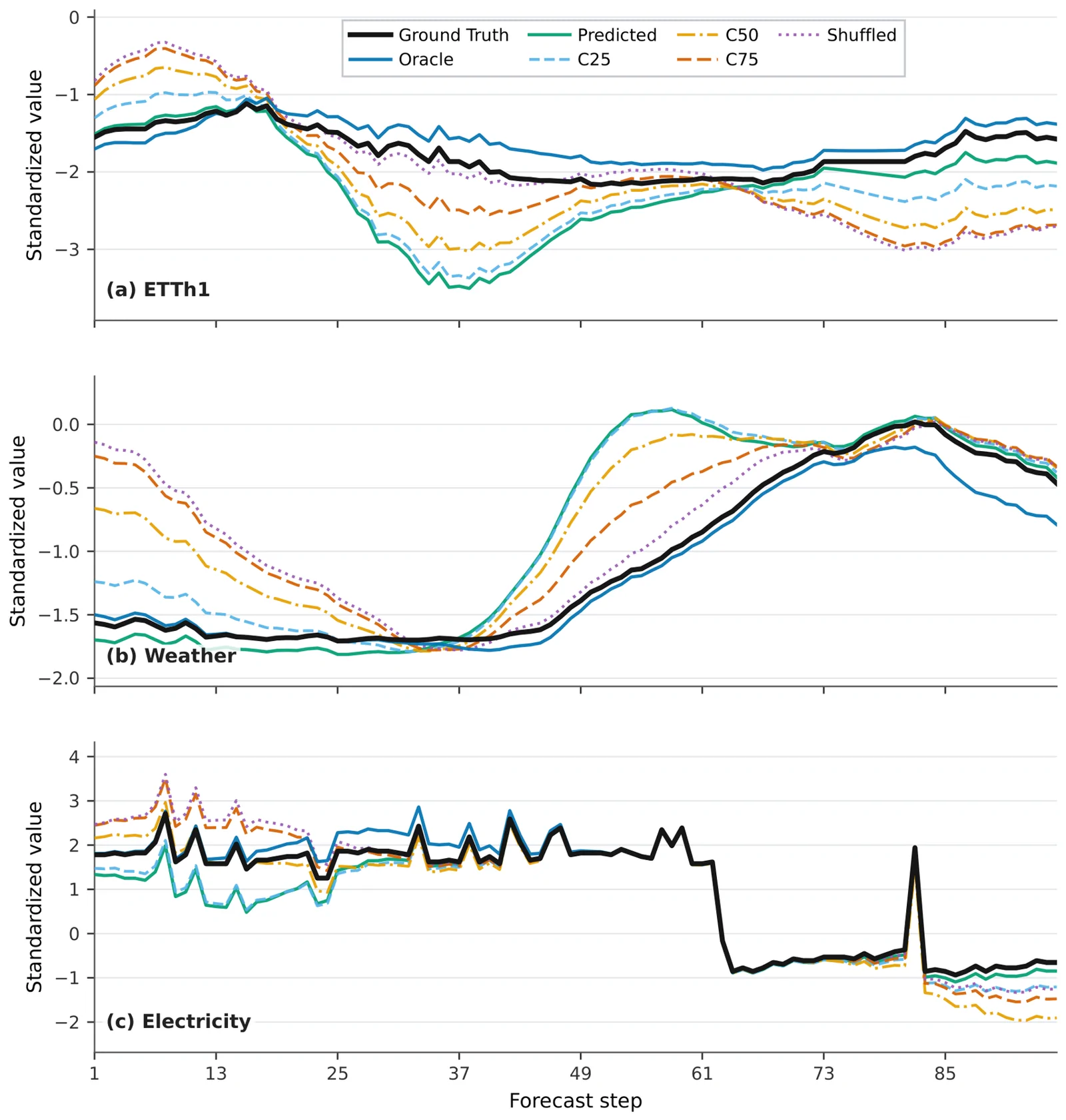
Illustrative forecast trajectories for one real standardized test period from ETTh1, Weather, and Electricity.

Figure 3 complements the aggregate results with a trajectory-level interpretation. A more accurate future pattern helps the forecast to retain the local level and turning behavior of the target. As the pattern is progressively corrupted, deviations in level and timing become more pronounced and persist over longer parts of the horizon.

The aligned predicted pattern provides more useful guidance than a pattern drawn from another sample. Predicted achieves a lower MSE and MAE than Shuffled on all nine datasets. At the macro level, shuffling increases the MSE from 0.2896 to 0.3305 and the MAE from 0.3211 to 0.3637. Equivalently, restoring the correct sample correspondence reduces the two errors by 12.37% and 11.72%, respectively. This comparison keeps the forecasting model and the set of predicted patterns fixed while changing their assignment to samples. It therefore shows the downstream value of the correspondence between the predicted pattern and the current history.

The intermediate corruption levels reveal that this dependence is graded rather than binary. The macro errors follow the ordered sequence of Predicted, C25, C50, C75, and Shuffled. Relative to Predicted, C25 raises the MSE and MAE by 3.32% and 3.15%; the increases reach 7.65% and 5.61% at C50 and 12.76% and 11.21% at C75. The response is not a fixed penalty that is proportional to the mixing coefficient. By C75, 90.39% of the complete MSE gap and 84.43% of the complete MAE gap between Predicted and Shuffled have already appeared. This concentration indicates that, once most of the sample-aligned change is removed, the remaining pattern carries only limited guidance for the decoder. The future pattern therefore acts as a graded information interface whose forecasting value depends on how much of the aligned structure it retains.

Oracle measures how the decoder uses a representation derived from the target itself. Its persistence and change inputs together determine $C_{Y}^{★}$. Under the Haar definition in Equation (5), the first detail component also determines successive value changes. Omitting the batch index, the future values satisfy

(36) $$Y\left[h,n\right]=X\left[L,n\right]-\sqrt{2}\sum_{r=1}^{h}C_{Y}^{★}\left[r,n,1\right],\;h=1,\ldots ,H.$$

Thus, the Oracle representation and the observed boundary value contain enough information to reconstruct the target. Its macro errors of 0.0382 in the MSE and 0.1308 in the MAE describe the decoder’s use of this target information. The Oracle–Predicted gap reflects the effect of replacing estimated patterns with target-derived patterns. How much of this gap can be closed depends on the future structure that is recoverable from the history. The direct pattern errors in Table 3 quantify the accuracy of estimates obtained from history. The Predicted–Shuffled comparison and corruption experiment then show how their sample alignment affects value forecasting. Together, these experiments connect the pattern estimation accuracy with its role in the forecasting model.

### 5.4. Pattern Ablations

The preceding analysis shows that the forecasting performance depends on the alignment and quality of the predicted future pattern. We next examine which properties make this pattern informative. The ablations are organized around two questions: what the pattern path should represent and how its evolution should be estimated from history. In Panel A of Table 5, History Only forecasts from the historical representation without a future pattern input. Static Pattern retains the last observed multiscale state but does not estimate its subsequent change. Raw Pattern replaces the multiscale decomposition with the identity mapping. It therefore represents future evolution directly as the raw value change from the last observation, corresponding to a single observation scale. The remaining pattern prediction, decoding, and training procedures are unchanged. Panel B isolates the role of frequency information. The Time Domain variant predicts the same multiscale future pattern using only the centered historical components and their temporal differences, without the frequency-filtered view. The decoder and training procedure remain unchanged.

Panel A first examines the value of representing future pattern evolution explicitly. Full obtains macro errors of 0.2896 in the MSE and 0.3211 in the MAE, compared with 0.3119 and 0.3563 for History Only. These differences correspond to reductions of 7.14% and 9.88%, respectively, and Full performs better on eight of the nine datasets for both metrics. Both variants receive the same historical observations. The improvement supports the use of separate pattern supervision to organize these observations into an estimate of future structural change for the decoder.

The comparisons with Static Pattern and Raw Pattern further identify which information is useful in this path. Relative to Static Pattern, Full reduces the macro MSE by 7.01% and the MAE by 4.71%, with seven wins in each metric. Relative to Raw Pattern, the reductions are 6.88% and 4.53%, with six wins in each metric. A repeated boundary state describes where the observed pattern ends, but it does not describe how the state changes across the forecasting horizon. A single pattern scale captures part of this change, whereas the complete representation preserves evolution with different temporal resolutions. Their aggregate differences show that the decoder benefits the most when the future change is both dynamic and multiscale.

We further compare EFP with an ordinary preliminary forecast in Table 6. This control, which is named Value Forecast, first learns to predict future values from history. Its predictor is then frozen, and the historical encoder and decoder are trained to produce the final forecast using these preliminary values. EFP instead supplies the decoder with the persistence state and predicted multiscale change. The two models use comparable parameter counts and follow the same data splits, lookback settings, forecasting horizons, and three runs, with the same two stages of training. This comparison examines the intermediate prediction target under comparable model capacity and training conditions.

EFP reduces the macro MSE from 0.2991 to 0.2896 and the macro MAE from 0.3331 to 0.3211, corresponding to reductions of 3.16% and 3.59%. It achieves lower errors on seven of the nine datasets for each metric. The differences vary across datasets: Value Forecast has a lower MSE on ETTm2 and Solar, while EFP has a lower MAE on both. On Traffic, EFP reduces the MSE, while the MAE values differ by just 0.0001. Thus, the effect of the intermediate target depends on both the dataset and the error measure. Across the evaluated datasets, the aggregate advantage supports multiscale future change as a useful intermediate target for value forecasting. This representation gives the decoder separate estimates of change at different temporal scales, extending the representation comparison in Panel A to an ordinary preliminary forecasting path.

Having compared the intermediate targets, Panel B examines how multiscale change should be estimated from history. The time-domain predictor produces macro errors of 0.3201 in the MSE and 0.3522 in the MAE, while the frequency-aware predictor reduces them to 0.2896 and 0.3211. This gives relative reductions of 9.53% and 8.83%, together with eight wins out of nine for each metric. The MSE improvements are especially visible on Weather, Solar, and Traffic, where temporal variation occurs over several characteristic rates. The filtered frequency view helps to identify stable and repeated evolution, while the centered and difference views retain level-dependent and local transitions. Their combination therefore provides a more informative estimate of future pattern change than time-domain evolution alone.

Together, the representation ablations, the Value Forecast comparison, and the predictor ablation support a coherent design path: the model explicitly predicts change beyond the observation boundary, preserves the change across multiple scales, and estimates it using complementary temporal and frequency information. These findings complement the quality analysis in Section 5.3. The earlier analysis links pattern accuracy and alignment to forecasting performance, while the present comparisons examine how the intermediate target and its predictor contribute to this performance. The next subsection examines how the forecasting model should learn from the errors that remain in the estimated pattern.

### 5.5. Training Strategy Ablations

The pattern available during training can be more accurate than the prediction used at inference. We therefore examine how the pattern sources used to train the forecasting model affect its performance when deployment relies on predicted patterns. Table 7 compares four strategies. Oracle Only trains the decoder with the supervised future pattern, while Predicted Only uses the output of the frozen pattern predictor. Oracle + Predicted combines these two sources. Mixed Strategy additionally includes Perturbed Oracle patterns constructed with structured prediction residuals. Its shuffled epoch schedule assigns Oracle, Predicted, and Perturbed Oracle to batches in proportions of 0.25, 0.50, and 0.25, respectively. All four strategies are evaluated with predicted future patterns at inference.

The two single-source strategies reveal complementary training roles. Oracle Only provides an accurate description of future pattern evolution and obtains macro errors of 0.3208 in the MSE and 0.3521 in the MAE. Predicted Only exposes the forecasting model to the patterns available at deployment and reduces these errors to 0.3139 and 0.3513. The first source gives a clear relation between pattern evolution and future values, while the second presents the estimation errors that the decoder encounters at inference. Their results motivate learning from both accurate patterns and deployment patterns.

Combining Oracle and Predicted improves the macro errors further to 0.3078 in the MSE and 0.3388 in the MAE. This result shows that accurate pattern supervision remains useful when it is paired with the predicted patterns used at deployment. The two sources represent the accurate target and the current predictor output as two discrete training states. Mixed Strategy broadens the training distribution with Perturbed Oracle patterns whose deviations follow structured prediction residuals.

Mixed Strategy achieves the lowest macro errors, with 0.2896 in the MSE and 0.3211 in the MAE. Relative to Oracle Only, it reduces the MSE by 9.72% and the MAE by 8.80%, with seven and eight dataset wins, respectively. Relative to Predicted Only, the reductions are 7.73% and 8.60%, and Mixed Strategy wins in both metrics on eight of the nine datasets. It also improves over Oracle + Predicted by 5.90% in the MSE and 5.21% in the MAE, with seven MSE wins and six MAE wins. These aggregate improvements show the value of combining accurate and predicted patterns with plausible structured perturbations.

The three pattern sources play complementary roles in this training process. Oracle anchors the mapping from future pattern evolution to target values, Predicted aligns training with the pattern available at inference, and Perturbed Oracle exposes the decoder to different levels of structured estimation error. Their mixture allows the forecasting model to use pattern changes across a range of accuracy levels. Together with the alignment and pattern design results, this ablation supports the training of the decoder to use the imperfect estimates available at deployment.

### 5.6. Computational Cost

Table 8 complements the accuracy results with the computational costs on ETTh1, Electricity, and Traffic. The measurements used a single NVIDIA GeForce RTX 4090 GPU, FP32 precision, and H=96, with the lookback lengths listed in the table. Inference latency is measured with a batch size of one. Peak memory refers to allocated GPU memory during training. The total parameter count includes the pattern predictor, historical encoder, and decoder, including parameters that are frozen in Stage 2.

All three configurations contain fewer than 0.86 million parameters. Electricity and Traffic have similar parameter counts because most weights are shared across variables, while their training memory peaks are 6 and 18 GiB, respectively. This distinction separates the model size from the cost of processing variable relations. The measured inference latency ranges from approximately 5.0 to 7.1 ms per sample for these settings.

## 6. Discussion

The experiments support future pattern evolution as a useful intermediate prediction in multivariate forecasting. EFP processes the same historical observations through two paths: one summarizes observed behavior, and the other estimates future multiscale change under separate supervision. The mechanism results show how the decoder uses this representation. Predicted patterns outperform shuffled patterns on all nine datasets, and the forecasting error rises in aggregate as sample alignment is weakened. The pattern and training ablations further favor dynamic multiscale change and exposure to realistic estimation errors on average. These results connect the value of the pattern path to its sample correspondence and to the way in which the decoder is trained to use it.

The target definition clarifies when explicit evolution is useful. If the multiscale state remains constant, its change from persistence is zero. Predicting change becomes relevant when the state evolves and the historical window contains cues about this evolution. Slow movement, repeated behavior, and local transitions can provide such cues at different scales. The learned gates allow their contributions to vary across samples and variables. This connects the usefulness of the pattern path to the predictability of change, rather than to variation alone.

The connection to prior work concerns both the representation and its use. Multiscale predictors organize forecasts across resolutions, while alignment and future teacher methods shape the learned representation through additional supervision [8,11,13,25]. EFP connects a fixed future change target to a separate predictor whose output remains in the value decoder at inference. Mixed training then links the target’s meaning to the errors of this predictor. This combination gives the intermediate prediction a defined role throughout training and deployment.

Oracle and Predicted serve different explanatory roles. Equation (36) shows that Oracle carries a representation from which the target can be recovered using the observed boundary value. Its low error therefore describes decoder behavior with target information. Predicted uses a pattern estimated from history, and its advantage over Shuffled supports the practical importance of sample correspondence. The amount of future change recoverable from history remains a question about the pattern predictor’s own error. This distinction is important because time series differ in complexity and predictability [41,42]. Similar historical windows can precede several plausible futures, so improvements in pattern estimation and the representation of uncertainty are connected research directions.

The ablations further show that the best pattern design is not identical for every series and metric. The complete model gives the best macro average, but simpler variants remain competitive in individual cases. In the pattern evolution ablation, the time-domain variant obtains a lower MSE on ETTm2 and a lower MAE on Traffic, whereas the frequency-aware model performs better on the other eight datasets for each metric. These local reversals are informative. Frequency information provides a strong overall prior, but a simpler temporal description can be sufficient when the relevant evolution is mainly local or when a metric gives more weight to a different error profile. The same principle applies to the multiscale representation: explicit evolution improves the aggregate result, while the relative value of individual scales depends on the data. This supports adaptive scale and frequency selection as a natural extension of the present fixed design.

The computational structure also defines a practical boundary. The future pattern predictor shares its parameters across variables. For a fixed lookback length, horizon, and hidden size, the main computation of this path therefore grows approximately linearly with the number of variables. The historical dependency encoder forms pairwise variable relations, so this part grows quadratically with the number of variables and is the main scaling concern for very high-dimensional data. The two-stage procedure also adds a separate pattern training stage, although inference uses only the historical input and a single predicted pattern. Sparse relation graphs, grouped variables, or approximate dependency masks can reduce this cost without changing the future pattern interface. This separation is useful because the pattern definition, pattern predictor, and dependency encoder can be improved independently.

The present formulation operates on regularly sampled windows. Missing observations affect both the historical decomposition and the persistence reference, especially when the last observation is missing. An imputed boundary value would propagate into every future reference step. A useful extension would combine imputation with an observation mask so that the predictor can distinguish measured values from estimates. Irregular sampling poses a different issue: the same index dilation can span different amounts of elapsed time. Resampling or filters defined over elapsed time would be needed to preserve the meaning of each scale. The current benchmark results concern the regularly sampled setting.

External shocks affect predictability even when sampling is regular. The predictor can learn transitions that leave cues in the observed history, but an event with no historical precursor requires another source of evidence. Weather forecasts, operating schedules, or event indicators could enter the pattern predictor as external variables when they are available at the forecast origin. This would retain the persistence and change interface while extending the information available for estimating future evolution.

The current decoder produces a point forecast from a single estimated pattern. To represent several plausible futures, a probabilistic pattern predictor could generate a distribution of multiscale changes given the history. Passing sampled changes through the decoder would produce alternative forecast trajectories. A probabilistic decoder could further represent the value uncertainty remaining after the pattern is specified. Prediction intervals derived from these outputs would require an evaluation of the coverage and width across horizons. They address uncertainty in future observations, whereas the standard deviations across repeated runs describe the variation in evaluation scores.

These extensions preserve the central idea of a defined intermediate prediction. Adaptive scales can change how evolution is represented, external variables can improve its estimation, and distributions over patterns can express alternative futures. The explicit interface makes these choices accessible for separate measurement while retaining their connection to the final forecast.

## 7. Conclusions

We studied the structural evolution that occurs after the observation boundary in multivariate time series forecasting. EFP represents this evolution as the multiscale change from the last observed pattern. Two coordinated paths process the historical input. A historical encoder summarizes the observed temporal behavior and variable relations, while a pattern predictor estimates the change over the forecast horizon. The decoder combines the two representations to produce future values.

The pattern path is trained through a direct future pattern target. The mixed training strategy then exposes the decoder to supervised patterns, predicted patterns, and structured perturbations. It learns how an accurate pattern relates to future values and how to use estimates with prediction errors. Future observations provide supervision during training; deployment requires only the historical input.

Experiments on nine datasets and four forecasting horizons support this formulation. The baseline comparisons assessed the forecasting accuracy across different model families, while the mechanism experiments examined how explicit future patterns guide prediction. The aligned predicted pattern outperformed the shuffled pattern on all nine datasets. The forecasting error also increases in aggregate as sample alignment is progressively weakened. Pattern ablations favor dynamic multiscale evolution on average, while frequency information improves the macro result across datasets. Training ablations further show that combining accurate, predicted, and perturbed patterns provides the strongest deployment performance on average among the compared strategies.

These results provide evidence that future pattern evolution can serve as a useful interface between historical representation and future value prediction. The interface connects separately supervised pattern estimation to its downstream forecasting value. Adaptive scales, probabilistic patterns, and sparse variable relations offer ways to extend this design. More broadly, an explicit future pattern allows its alignment, quality, uncertainty, and forecasting value to be studied separately. This provides a structured basis for improving multivariate forecasting beyond a single direct mapping from past values to future values.

## Figures and Tables

**Table 1:** Statistical information for the nine multivariate time series datasets. Dataset Size gives the numbers of samples in the training, validation, and test splits, respectively.

Dataset	Dim.	Prediction Length	Dataset Size (Train, Validation, Test)	Frequency
ETTh1	7	{96,192,336,720}	(8545,2881,2881)	1 h
ETTh2	7	{96,192,336,720}	(8545,2881,2881)	1 h
ETTm1	7	{96,192,336,720}	(34,465,11,521,11,521)	15 min
ETTm2	7	{96,192,336,720}	(34,465,11,521,11,521)	15 min
Exchange	8	{96,192,336,720}	(5120,665,1422)	1 day
Weather	21	{96,192,336,720}	(36,792,5271,10,540)	10 min
Electricity	321	{96,192,336,720}	(18,317,2633,5261)	1 h
Solar-Energy	137	{96,192,336,720}	(36,601,5161,10,417)	10 min
Traffic	862	{96,192,336,720}	(12,185,1757,3509)	1 h

**Table 2:** Multivariate time series forecasting results on nine datasets under four prediction horizons. Lower values indicate better performance. Mean results are shown here; mean ± SD is provided in Appendix E Table A5. The best result is shown in **bold red**, and the second-best result is shown in underlined blue. The final two rows report the numbers of first-place results for MSE and MAE, respectively.

Dataset	*H*	EFP (Ours)		iTransformer		TimeMixer		PatchTST		Crossformer		TimesNet		DLinear	
		MSE	MAE	MSE	MAE	MSE	MAE	MSE	MAE	MSE	MAE	MSE	MAE	MSE	MAE
ETTh1	96	0.381	0.401	0.386	0.405	**0.372**	0.401	0.377	**0.397**	0.411	0.435	0.389	0.412	0.379	0.403
	192	**0.405**	0.429	0.424	0.440	0.413	0.430	0.409	0.425	0.409	0.438	0.440	0.443	0.408	**0.419**
	336	**0.428**	**0.436**	0.449	0.460	0.438	0.450	0.431	0.444	0.433	0.457	0.523	0.487	0.440	0.440
	720	**0.451**	**0.472**	0.495	0.487	0.486	0.484	0.457	0.477	0.501	0.514	0.521	0.495	0.471	0.493
	Avg.	**0.416**	**0.435**	0.439	0.448	0.427	0.441	0.419	0.436	0.439	0.461	0.468	0.459	0.425	0.439
ETTh2	96	**0.274**	0.341	0.297	0.348	0.281	0.351	0.277	**0.337**	0.728	0.603	0.334	0.370	0.300	0.364
	192	**0.346**	0.387	0.372	0.403	0.349	0.389	0.348	**0.384**	0.723	0.607	0.404	0.413	0.387	0.423
	336	**0.363**	**0.410**	0.388	0.417	0.366	0.413	0.377	0.416	0.740	0.628	0.389	0.435	0.490	0.487
	720	**0.396**	**0.432**	0.424	0.444	0.401	0.436	0.406	0.441	1.386	0.882	0.434	0.448	0.704	0.597
	Avg.	**0.345**	**0.393**	0.370	0.403	0.349	0.397	0.352	0.395	0.894	0.680	0.390	0.417	0.470	0.468
ETTm1	96	**0.287**	**0.342**	0.300	0.353	0.293	0.345	0.289	0.343	0.314	0.367	0.340	0.378	0.300	0.345
	192	**0.325**	**0.366**	0.341	0.380	0.335	0.372	0.329	0.368	0.374	0.410	0.392	0.404	0.336	0.369
	336	**0.359**	0.387	0.374	0.396	0.368	0.388	0.362	0.390	0.413	0.432	0.423	0.426	0.367	**0.386**
	720	**0.412**	**0.410**	0.429	0.430	0.426	0.417	0.416	0.423	0.753	0.613	0.475	0.453	0.419	0.416
	Avg.	**0.346**	**0.376**	0.361	0.390	0.356	0.381	0.349	0.381	0.464	0.456	0.408	0.415	0.356	0.379
ETTm2	96	**0.162**	**0.249**	0.175	0.266	0.165	0.256	0.165	0.255	0.296	0.391	0.189	0.265	0.164	0.255
	192	**0.219**	**0.291**	0.242	0.312	0.225	0.298	0.221	0.293	0.369	0.416	0.254	0.310	0.224	0.304
	336	**0.273**	**0.324**	0.282	0.337	0.277	0.332	0.276	0.327	0.588	0.600	0.313	0.345	0.277	0.337
	720	**0.356**	**0.378**	0.375	0.394	0.360	0.387	0.362	0.381	0.750	0.612	0.413	0.402	0.371	0.401
	Avg.	**0.253**	**0.311**	0.269	0.327	0.257	0.318	0.256	0.314	0.501	0.505	0.292	0.331	0.259	0.324
Exchange	96	0.080	**0.200**	0.086	0.205	0.084	0.207	**0.079**	0.202	0.088	0.213	0.112	0.242	0.080	0.202
	192	0.161	**0.287**	0.177	0.299	0.178	0.300	0.159	0.289	**0.157**	0.288	0.209	0.334	0.182	0.321
	336	**0.296**	**0.396**	0.331	0.417	0.376	0.451	0.297	0.399	0.332	0.429	0.358	0.435	0.327	0.434
	720	**0.581**	**0.603**	0.846	0.693	0.884	0.707	0.751	0.650	0.980	0.762	0.944	0.736	0.583	0.605
	Avg.	**0.280**	**0.372**	0.360	0.404	0.381	0.416	0.322	0.385	0.389	0.423	0.406	0.437	0.293	0.391
Weather	96	0.144	0.198	0.157	0.207	0.147	0.201	0.149	**0.196**	**0.143**	0.210	0.168	0.214	0.170	0.230
	192	**0.190**	**0.237**	0.200	0.248	0.192	0.243	0.191	0.239	0.198	0.260	0.219	0.262	0.216	0.273
	336	**0.239**	**0.276**	0.252	0.287	0.247	0.284	0.242	0.279	0.258	0.314	0.278	0.302	0.258	0.307
	720	**0.311**	**0.322**	0.320	0.336	0.318	0.330	0.313	0.330	0.335	0.385	0.353	0.351	0.323	0.362
	Avg.	**0.221**	**0.258**	0.232	0.270	0.226	0.265	0.224	0.261	0.234	0.292	0.255	0.282	0.242	0.293
Electricity	96	**0.130**	**0.225**	0.134	0.230	0.153	0.256	0.143	0.247	0.134	0.231	0.169	0.271	0.140	0.237
	192	**0.146**	**0.241**	0.154	0.250	0.168	0.269	0.158	0.260	0.147	0.243	0.180	0.280	0.154	0.251
	336	**0.165**	**0.262**	0.169	0.265	0.189	0.291	0.168	0.267	**0.165**	0.264	0.204	0.304	0.169	0.268
	720	**0.195**	**0.289**	0.197	0.292	0.228	0.320	0.214	0.307	0.237	0.314	0.205	0.304	0.204	0.301
	Avg.	**0.159**	**0.254**	0.164	0.259	0.185	0.284	0.171	0.270	0.171	0.263	0.190	0.290	0.167	0.264
Solar	96	**0.170**	**0.204**	0.190	0.244	0.179	0.232	0.172	0.234	0.183	0.208	0.198	0.270	0.199	0.265
	192	**0.191**	**0.221**	0.193	0.257	0.201	0.259	0.204	0.302	0.208	0.226	0.206	0.276	0.220	0.282
	336	0.201	**0.231**	0.203	0.266	**0.190**	0.256	0.212	0.293	0.212	0.239	0.208	0.284	0.234	0.295
	720	0.211	**0.244**	0.223	0.281	**0.203**	0.261	0.215	0.307	0.215	0.256	0.232	0.294	0.243	0.301
	Avg.	**0.193**	**0.225**	0.202	0.262	**0.193**	0.252	0.201	0.284	0.205	0.232	0.211	0.281	0.224	0.286
Traffic	96	**0.362**	**0.250**	0.364	0.265	0.369	0.257	0.370	0.262	0.526	0.288	0.595	0.312	0.395	0.275
	192	0.385	**0.259**	**0.384**	0.273	0.400	0.272	0.386	0.269	0.503	0.263	0.613	0.322	0.407	0.280
	336	**0.396**	**0.271**	0.398	0.277	0.407	0.272	**0.396**	0.275	0.505	0.276	0.626	0.332	0.417	0.286
	720	**0.435**	**0.289**	0.445	0.308	0.461	0.316	**0.435**	0.295	0.552	0.301	0.635	0.340	0.454	0.308
	Avg.	**0.395**	**0.267**	0.398	0.281	0.409	0.279	0.397	0.275	0.522	0.282	0.617	0.327	0.418	0.287
MSE Best Count		29		1		3		3		3		0		0	
MAE Best Count		30		0		0		4		0		0		2	

**Table 3:** Test errors of future pattern change estimates. Each dataset entry averages four forecasting horizons and three runs. Average denotes the arithmetic mean across the nine datasets. Lower values indicate better performance, and the better result for each metric is shown in bold.

Dataset	Persistence MSE	Persistence MAE	Predictor MSE	Predictor MAE
ETTh1	0.1974	0.3031	**0.1466**	**0.2167**
ETTh2	0.2616	0.3264	**0.1809**	**0.2668**
ETTm1	0.1569	0.2562	**0.1167**	**0.2004**
ETTm2	0.1118	0.1919	**0.0786**	**0.1576**
Exchange	0.3427	0.3761	**0.2978**	**0.3611**
Weather	0.1435	0.2276	**0.1188**	**0.2056**
Electricity	0.0982	0.1797	**0.0936**	**0.1631**
Solar	0.2273	0.2972	**0.1695**	**0.2598**
Traffic	0.0861	0.1714	**0.0628**	**0.1536**
Average	0.1806	0.2588	**0.1406**	**0.2205**

**Table 4:** Forecasting errors under different future pattern sources and corruption levels. Each dataset entry averages the four forecasting horizons. Oracle is a diagnostic pattern obtained from the true future sequence. C25, C50, and C75 denote corruption levels ρ=0.25, 0.50, and 0.75 in Equation (14). Lower values indicate better performance.

Dataset	Oracle		Predicted		C25		C50		C75		Shuffled	
	MSE	MAE	MSE	MAE	MSE	MAE	MSE	MAE	MSE	MAE	MSE	MAE
ETTh1	0.045	0.215	0.416	0.435	0.421	0.462	0.436	0.455	0.462	0.478	0.455	0.496
ETTh2	0.068	0.166	0.345	0.393	0.350	0.409	0.352	0.425	0.359	0.437	0.362	0.451
ETTm1	0.047	0.198	0.346	0.376	0.357	0.383	0.370	0.390	0.391	0.400	0.406	0.403
ETTm2	0.030	0.097	0.253	0.311	0.268	0.328	0.284	0.328	0.300	0.351	0.300	0.366
Exchange	0.032	0.155	0.280	0.372	0.283	0.377	0.297	0.393	0.316	0.419	0.311	0.416
Weather	0.018	0.095	0.221	0.258	0.230	0.263	0.234	0.272	0.248	0.306	0.245	0.306
Electricity	0.038	0.076	0.159	0.254	0.169	0.261	0.178	0.276	0.186	0.284	0.200	0.285
Solar	0.020	0.084	0.193	0.225	0.208	0.228	0.231	0.231	0.226	0.251	0.249	0.250
Traffic	0.045	0.091	0.395	0.267	0.407	0.270	0.424	0.282	0.451	0.288	0.447	0.302
Average	**0.0382**	**0.1308**	**0.2896**	**0.3211**	**0.2992**	**0.3312**	**0.3118**	**0.3391**	**0.3266**	**0.3571**	**0.3305**	**0.3637**

**Table 5:** Ablations of future pattern representation and prediction. All variants are trained independently under the same experimental protocol. The Time Domain predictor is also trained independently. It removes the frequency-filtered view while retaining the centered historical components, temporal differences, decoder, and mixed training strategy. Lower values indicate better performance, and the best result for each dataset and metric is shown in bold.

**Panel A: Future Pattern Representation**								
**Dataset**	**History Only**		**Static Pattern**		**Raw Pattern**		**Full**	
	**MSE**	**MAE**	**MSE**	**MAE**	**MSE**	**MAE**	**MSE**	**MAE**
ETTh1	**0.4110**	0.4780	**0.4110**	0.4540	0.4350	0.4570	0.4163	**0.4345**
ETTh2	0.3490	0.4230	0.3590	0.3970	**0.3380**	**0.3810**	0.3448	0.3925
ETTm1	0.4080	0.4230	0.4080	**0.3760**	0.4050	0.4230	**0.3458**	0.3763
ETTm2	0.2670	0.3630	0.2820	0.3190	0.2560	**0.2970**	**0.2525**	0.3105
Exchange	0.3430	0.4250	**0.2640**	0.3990	0.3020	0.3740	0.2795	**0.3715**
Weather	0.2220	0.2800	0.2470	0.3250	**0.2130**	0.2740	0.2210	**0.2583**
Electricity	0.1900	0.2990	0.1860	0.2600	**0.1590**	**0.2390**	**0.1590**	0.2543
Solar	0.2000	0.2630	0.2380	0.2440	0.2400	0.2820	**0.1933**	**0.2250**
Traffic	0.4170	**0.2530**	0.4080	0.2590	0.4510	0.3000	**0.3945**	0.2673
Average	0.3119	0.3563	0.3114	0.3370	0.3110	0.3363	**0.2896**	**0.3211**
**Panel B: Pattern Evolution**								
**Dataset**	**Time Domain**				**Frequency Aware**			
	**MSE**		**MAE**		**MSE**		**MAE**	
ETTh1	0.4430		0.4400		**0.4163**		**0.4345**	
ETTh2	0.3500		0.4100		**0.3448**		**0.3925**	
ETTm1	0.3670		0.4160		**0.3458**		**0.3763**	
ETTm2	**0.2480**		0.3830		0.2525		**0.3105**	
Exchange	0.2840		0.4340		**0.2795**		**0.3715**	
Weather	0.2660		0.2900		**0.2210**		**0.2583**	
Electricity	0.1840		0.2740		**0.1590**		**0.2543**	
Solar	0.2490		0.2600		**0.1933**		**0.2250**	
Traffic	0.4900		**0.2630**		**0.3945**		0.2673	
Average	0.3201		0.3522		**0.2896**		**0.3211**	

**Table 6:** Comparison with ordinary preliminary value forecasting under comparable model capacity. Each dataset entry reports the mean ± SD across three runs after averaging the four forecasting horizons. Average denotes the arithmetic mean of the displayed means across the nine datasets. Lower values indicate better performance, and the better result for each metric is shown in **bold red**.

Dataset	Value Forecast		EFP	
	MSE	MAE	MSE	MAE
ETTh1	0.4225 ± 0.0029	0.4586 ± 0.0016	**0.4163 ± 0.0048**	**0.4345 ± 0.0023**
ETTh2	0.3523 ± 0.0033	0.4011 ± 0.0064	**0.3448 ± 0.0072**	**0.3925 ± 0.0073**
ETTm1	0.3546 ± 0.0042	0.3927 ± 0.0024	**0.3458 ± 0.0017**	**0.3763 ± 0.0044**
ETTm2	**0.2453 ± 0.0042**	0.3146 ± 0.0084	0.2525 ± 0.0056	**0.3105 ± 0.0068**
Exchange	0.3071 ± 0.0051	0.3924 ± 0.0079	**0.2795 ± 0.0053**	**0.3715 ± 0.0045**
Weather	0.2269 ± 0.0029	**0.2525 ± 0.0044**	**0.2210 ± 0.0038**	0.2583 ± 0.0054
Electricity	0.1663 ± 0.0035	0.2675 ± 0.0048	**0.1590 ± 0.0055**	**0.2543 ± 0.0066**
Solar	**0.1899 ± 0.0015**	0.2513 ± 0.0033	0.1933 ± 0.0059	**0.2250 ± 0.0062**
Traffic	0.4269 ± 0.0086	**0.2672 ± 0.0078**	**0.3945 ± 0.0047**	0.2673 ± 0.0069
Average	0.2991	0.3331	**0.2896**	**0.3211**

**Table 7:** Ablations of the training strategy, evaluated with predicted future patterns at inference. Mixed Strategy assigns Oracle, Predicted, and Perturbed Oracle patterns to batches in proportions of 0.25, 0.50, and 0.25. Each dataset entry reports the mean ± SD across three runs after averaging the four forecasting horizons. Average denotes the arithmetic mean of the displayed means across datasets. Lower values indicate better performance, and the best result in each row is shown in **bold red**.

Dataset	Oracle Only		Predicted Only		Oracle + Predicted		Mixed Strategy	
	MSE	MAE	MSE	MAE	MSE	MAE	MSE	MAE
ETTh1	0.4470 ± 0.0034	0.4470 ± 0.0061	0.4220 ± 0.0053	0.4480 ± 0.0027	0.4170 ± 0.0043	**0.4300 ± 0.0060**	**0.4163 ± 0.0014**	0.4345 ± 0.0055
ETTh2	0.3890 ± 0.0068	0.3860 ± 0.0025	0.3890 ± 0.0024	0.3810 ± 0.0038	0.3680 ± 0.0083	**0.3790 ± 0.0034**	**0.3448 ± 0.0071**	0.3925 ± 0.0036
ETTm1	0.3470 ± 0.0035	0.4120 ± 0.0040	0.3870 ± 0.0075	0.4260 ± 0.0045	**0.3390 ± 0.0022**	**0.3640 ± 0.0083**	0.3458 ± 0.0053	0.3763 ± 0.0041
ETTm2	0.3100 ± 0.0066	0.3320 ± 0.0054	0.2620 ± 0.0089	0.3410 ± 0.0066	0.2760 ± 0.0067	0.3200 ± 0.0024	**0.2525 ± 0.0059**	**0.3105 ± 0.0064**
Exchange	0.2730 ± 0.0054	0.4190 ± 0.0041	0.2710 ± 0.0034	0.4350 ± 0.0055	**0.2690 ± 0.0069**	0.4120 ± 0.0036	0.2795 ± 0.0039	**0.3715 ± 0.0048**
Weather	**0.2150 ± 0.0051**	0.3040 ± 0.0053	0.2630 ± 0.0023	0.3140 ± 0.0063	0.2560 ± 0.0047	0.2990 ± 0.0035	0.2210 ± 0.0042	**0.2583 ± 0.0030**
Electricity	0.1960 ± 0.0048	0.2930 ± 0.0065	0.2100 ± 0.0041	0.3090 ± 0.0050	0.1780 ± 0.0062	0.2970 ± 0.0070	**0.1590 ± 0.0046**	**0.2543 ± 0.0082**
Solar	0.2200 ± 0.0020	0.2740 ± 0.0017	0.2080 ± 0.0046	0.2340 ± 0.0044	0.2480 ± 0.0045	0.2400 ± 0.0059	**0.1933 ± 0.0042**	**0.2250 ± 0.0047**
Traffic	0.4900 ± 0.0074	0.3020 ± 0.0052	0.4130 ± 0.0079	0.2740 ± 0.0027	0.4190 ± 0.0045	0.3080 ± 0.0068	**0.3945 ± 0.0068**	**0.2673 ± 0.0074**
Average	0.3208	0.3521	0.3139	0.3513	0.3078	0.3388	**0.2896**	**0.3211**

**Table 8:** Computational cost of EFP. Training times and inference latencies are approximate; parameter counts are exact.

Dataset	*L*	Parameters	Training Time (min)			Latency (ms/Sample)	Peak Memory (GiB)
			Stage 1	Stage 2	Total		
ETTh1	512	604,324	1	1	2	5.1	2.0
Electricity	720	856,112	25	40	65	5.0	6.0
Traffic	720	857,194	45	90	135	7.1	18.0

## Data Availability

All datasets analyzed in this study are publicly available through the TFB benchmark repository at https://github.com/decisionintelligence/TFB (accessed on 23 May 2026).

## Appendix A. Lookback Selection

We select the lookback separately for each dataset and method. The candidates are L∈{336,512,720}. The selection score is the mean validation MSE over horizons H∈{96,192,336,720}. The selected lookback is shared across these four horizons and the three runs with seeds 2024, 2025, and 2026. We train separate model weights for each horizon and seed. Table A1 lists the final choices. Test scores are reported for the selected configurations and are not used to choose *L*.

**Table A1:** Lookback lengths selected by mean validation MSE. Each entry applies to all four horizons and three repeated runs for the given method and dataset.

Dataset	EFP	iTransformer		TimeMixer	PatchTST	Crossformer	TimesNet	DLinear
ETTh1	512	512		512	336	512	512	336
ETTh2	336	512		336	336	512	336	512
ETTm1	512	512		512	512	512	512	512
ETTm2	512	336		336	512	512	512	512
Exchange	336	336		512	512	512	512	336
Weather	336	336		336	512	512	336	336
Electricity	720	512		512	720	720	720	512
Solar	336	336		336	336	336	336	336
Traffic	720	720		512	512	720	512	512
**Dataset**	**AMD**	**FITS**	**Pathformer**	**DUET**	**SimpleTM**	**ModernTCN**	**TimeBridge**	**MOIRAI**
ETTh1	512	336	512	512	512	336	512	336
ETTh2	336	336	336	512	336	336	512	512
ETTm1	512	512	336	512	512	512	336	512
ETTm2	512	512	720	512	512	336	512	512
Exchange	512	512	512	512	512	512	512	336
Weather	336	336	336	336	336	512	336	512
Electricity	720	512	720	336	512	336	512	336
Solar	512	512	336	512	512	512	512	720
Traffic	720	720	720	512	720	512	720	512

## Appendix B. EFP Implementation

Table A2 gives the EFP architecture settings. The pattern predictor shares its parameters across variables. These settings are fixed across the nine datasets; the selected *L*, forecasting horizon *H*, and number of variables determine the input and output dimensions.

**Table A2:** Architecture settings for EFP.

Setting	Value
Decomposition	Haar/db1, no downsampling, left replication
Levels/component order	J=4; $D_{1},D_{2},D_{3},D_{4},A_{4}$
Hidden/feedforward dimension	64/128
Variable attention layers/heads	1/4
Dropout/activation	0.1/GELU
Historical scale gate	4K→32→K, softmax output
Local normalization	Per sample and variable; $\epsilon =10^{-5}$; learned affine parameters; detached mean and population standard deviation
Pattern input projection	3K→64, with K=5
Pattern temporal blocks	Two depthwise blocks; kernel 5; dilations 1 and 2; left replication
Pattern block operations	Depthwise convolution, pointwise convolution, GroupNorm with one group, GELU, dropout, residual addition
Pattern temporal/output projection	L→H/64→K
Pattern linear branch	Separate L→H map per component; enabled
Pattern gates	Sigmoid frequency gate and component residual gate
Decoder pattern stream	2K→64; depthwise kernel 3, zero padding 1; pointwise kernel 1; residual addition and LayerNorm
Decoder fusion gate	128→64→64, sigmoid output
Forecast head	64→64→1, with GELU and dropout
Horizon embeddings	H×64; truncated normal initialization, standard deviation 0.02

The historical temporal encoder decomposes the locally normalized window. The pattern predictor and dependency mask use the dataset standardized window. For each variable pair, the dependency module transforms differences between historical magnitude spectra with a learned square metric of size F×F, where F=⌊L/2⌋+1. It takes the reciprocal squared distance with an offset of $10^{-10}$, removes the diagonal, and divides each row by its largest value. The diagonal is then set to one and all scores are multiplied by 0.99.

To sample the mask, the scores are clamped to $\left[10^{-6},1-10^{-6}\right]$. Each score *p* forms the two logits log(p/(1−p)) and log((1−p)/p). Hard Gumbel softmax with temperature 1 selects the binary mask entry. Sampling remains active during evaluation. These operations implement the historical dependency mask; the future pattern predictor has no Gumbel sampling.

## Appendix C. Training and Randomness

Table A3 gives the settings used in both stages. In Stage 1, only the pattern predictor is optimized. Stage 2 freezes it and optimizes the historical encoder and decoder. The frozen predictor stays in evaluation mode. In each stage, an epoch without a lower validation metric counts toward patience; the best checkpoint is restored after stopping.

**Table A3:** Training settings for EFP.

Setting	Value
Optimizer	AdamW in both stages
Learning rate/weight decay	$10^{-3}$/$10^{-4}$
Batch size	32 in both stages; retain the final partial batch
Maximum epochs/patience	300/50 per stage
Gradient clipping	Global norm threshold 1.0
Stage 1 loss	SmoothL1, transition parameter 1, mean reduction
Stage 1 selection	Lowest validation RMSE of pattern change
Stage 2 loss	Forecast MSE, mean reduction
Stage 2 selection	Lowest validation forecast MSE with predicted patterns
Stage 2 pattern mixture	Oracle 0.25; Predicted 0.50; Perturbed Oracle 0.25
Perturbation scale	Uniform choice from {0.5,1.0}, one value per batch
Perturbation residuals	Independently sampled training windows, with replacement
Joint refinement	Disabled
Run seeds	2024, 2025, 2026
Test evaluation seed	Run seed +500,001
Tensor precision	Float32
Hardware/framework	One NVIDIA GeForce RTX 4090 GPU/Python 3.13.13; NumPy 2.3.5; PyTorch 2.11.0

The Stage 2 source schedule rounds the specified proportions to whole batch counts and shuffles their order at each epoch. All samples in a batch use the same source. For Perturbed Oracle, an additional batch of training windows supplies prediction residuals. These residuals are added to the current batch’s true pattern changes with the sampled scale; the persistence reference is unchanged. Each run seeds Python 3.13.13, NumPy 2.3.5, and PyTorch 2.11.0. Data loading uses a seeded generator. In Stage 2, the source schedule and perturbation scale use a Python generator with seed offset 91,001, and residual window sampling uses a NumPy generator with offset 81,001. Before the final test evaluation, the random state is reset to the run seed plus 500,001 to control dependency mask sampling. Dropout is disabled during evaluation. Fixing this random state means that repeated evaluations of a checkpoint use the same sequence of sampled masks.

## Appendix D. Additional Baselines

We extend the comparison to AMD [27], FITS [16], Pathformer [19], DUET [20], SimpleTM [39], ModernTCN [17], TimeBridge [15], and MOIRAI [40]. We use their official implementations and evaluate MOIRAI in the zero-shot setting. This set includes adaptive multiscale models, convolutional architectures, and a pretrained probabilistic forecasting model. Table A4 reports the MSE and MAE for the same nine datasets and four forecasting horizons.

**Table A4:** Forecasting results for EFP and eight additional models on nine datasets. Lower MSE and MAE indicate better performance. Avg. is the mean over the four horizons, rounded to three decimal places. Bold and underlining indicate the best and second-best distinct values among the methods in this table, with ties marked equally.

Dataset	*H*	EFP		AMD		FITS		Pathformer		DUET		SimpleTM		ModernTCN		TimeBridge		MOIRAI	
		(Ours)		(2025)		(2024)		(2024)		(2025)		(2025)		(2024)		(2025)		(2024)	
		MSE	MAE	MSE	MAE	MSE	MAE	MSE	MAE	MSE	MAE	MSE	MAE	MSE	MAE	MSE	MAE	MSE	MAE
ETTh1	96	0.381	0.401	0.369	0.397	0.386	0.396	0.372	0.392	**0.352**	**0.384**	0.390	0.410	0.368	0.394	0.375	0.390	0.380	0.398
	192	0.405	0.429	0.407	0.416	0.436	0.423	0.408	0.415	**0.398**	**0.409**	0.422	0.428	0.405	0.413	0.428	0.421	0.440	0.434
	336	0.428	0.436	0.418	0.427	0.478	0.444	0.438	0.434	0.414	0.426	0.440	0.440	**0.391**	**0.412**	0.470	0.443	0.514	0.474
	720	0.451	0.472	0.439	**0.454**	0.502	0.495	0.450	0.463	**0.429**	0.455	0.459	0.475	0.450	0.461	0.481	0.481	0.705	0.568
	Avg.	0.416	0.435	0.408	0.424	0.451	0.440	0.417	0.426	**0.398**	**0.419**	0.428	0.438	0.404	0.420	0.439	0.434	0.510	0.469
ETTh2	96	0.274	0.341	0.274	0.337	0.295	0.350	0.279	0.336	0.276	0.336	0.298	0.349	**0.263**	0.332	0.286	0.333	0.287	**0.325**
	192	0.346	0.387	0.351	0.383	0.381	0.396	0.345	0.380	0.332	0.374	0.379	0.396	**0.320**	0.374	0.364	0.385	0.347	**0.367**
	336	0.363	0.410	0.375	0.411	0.426	0.438	0.378	0.408	0.353	0.397	0.400	0.420	**0.313**	**0.376**	0.416	0.425	0.377	0.393
	720	0.396	0.432	0.402	0.438	0.431	0.446	0.437	0.455	**0.382**	0.425	0.432	0.451	0.397	0.433	0.435	0.448	0.404	**0.421**
	Avg.	0.345	0.393	0.351	0.392	0.383	0.408	0.360	0.395	0.336	0.383	0.377	0.404	**0.323**	0.379	0.375	0.398	0.354	**0.377**
ETTm1	96	0.287	0.342	0.284	0.339	0.355	0.375	0.290	0.335	**0.279**	**0.333**	0.286	0.338	0.292	0.346	0.312	0.344	0.353	0.363
	192	0.325	0.366	0.328	0.362	0.392	0.393	0.337	0.363	**0.320**	**0.358**	0.326	0.364	0.332	0.368	0.366	0.377	0.376	0.380
	336	0.359	0.387	0.360	0.380	0.424	0.414	0.374	0.384	**0.348**	**0.377**	0.364	0.388	0.365	0.391	0.403	0.404	0.399	0.395
	720	0.412	0.410	0.421	0.416	0.487	0.449	0.428	0.416	**0.405**	**0.408**	0.424	0.426	0.416	0.417	0.454	0.438	0.432	0.417
	Avg.	0.346	0.376	0.348	0.374	0.415	0.408	0.357	0.375	**0.338**	**0.369**	0.350	0.379	0.351	0.381	0.384	0.391	0.390	0.389
ETTm2	96	0.162	0.249	0.167	0.258	0.183	0.266	0.164	0.250	**0.161**	**0.248**	0.174	0.257	0.166	0.256	0.175	0.252	0.189	0.260
	192	0.219	0.291	0.221	0.294	0.247	0.305	0.219	0.288	**0.214**	**0.286**	0.233	0.297	0.222	0.293	0.246	0.299	0.247	0.300
	336	0.273	0.324	0.270	0.327	0.307	0.342	**0.267**	**0.319**	**0.267**	0.321	0.279	0.330	0.272	0.324	0.311	0.341	0.295	0.334
	720	0.356	0.378	0.356	0.382	0.407	0.399	0.361	0.377	**0.348**	**0.374**	0.367	0.387	0.358	0.381	0.403	0.397	0.372	0.386
	Avg.	0.253	0.311	0.254	0.315	0.286	0.328	0.253	0.309	**0.248**	**0.307**	0.263	0.318	0.255	0.314	0.284	0.322	0.276	0.320
Exchange	96	**0.080**	0.200	0.083	0.201	0.089	0.202	0.088	0.208	**0.080**	**0.198**	0.093	0.211	0.095	0.206	0.091	0.214	0.090	0.204
	192	**0.161**	**0.287**	0.171	0.293	0.165	0.293	0.183	0.304	0.162	0.288	0.166	0.288	0.170	0.291	0.164	0.295	0.168	0.295
	336	0.296	0.396	0.309	0.402	0.298	0.397	0.354	0.429	**0.294**	**0.392**	0.308	0.408	0.319	0.402	0.306	0.400	0.302	0.405
	720	**0.581**	0.603	0.750	0.652	0.778	0.624	0.909	0.716	0.583	**0.580**	0.767	0.652	0.765	0.604	0.770	0.631	0.620	0.648
	Avg.	**0.280**	0.372	0.328	0.387	0.333	0.379	0.384	0.414	**0.280**	**0.365**	0.334	0.390	0.337	0.376	0.333	0.385	0.295	0.388
Weather	96	**0.144**	0.198	0.145	0.197	0.166	0.213	0.148	0.195	0.146	**0.191**	0.148	0.197	0.149	0.200	0.169	0.206	0.177	0.208
	192	0.190	0.237	**0.187**	0.238	0.213	0.254	0.191	0.235	0.190	**0.231**	0.195	0.244	0.196	0.245	0.219	0.249	0.219	0.249
	336	0.239	0.276	0.240	0.280	0.269	0.294	0.243	0.274	**0.234**	**0.268**	0.249	0.287	0.241	0.277	0.274	0.290	0.277	0.292
	720	0.311	0.322	0.315	0.330	0.346	0.343	0.318	0.326	**0.305**	**0.319**	0.329	0.339	0.314	0.334	0.353	0.341	0.365	0.350
	Avg.	0.221	0.258	0.222	0.261	0.249	0.276	0.225	0.258	**0.219**	**0.252**	0.230	0.267	0.225	0.264	0.254	0.272	0.260	0.275
Electricity	96	0.130	0.225	0.130	0.224	0.200	0.278	0.135	0.222	**0.128**	**0.219**	0.146	0.245	0.131	0.226	0.138	0.232	0.152	0.242
	192	0.146	0.241	0.147	0.238	0.200	0.280	0.157	0.253	0.148	**0.235**	0.164	0.262	**0.143**	0.239	0.157	0.251	0.171	0.259
	336	0.165	0.262	**0.160**	**0.253**	0.214	0.295	0.170	0.267	0.163	0.255	0.183	0.281	0.161	0.259	0.178	0.272	0.192	0.278
	720	0.195	0.289	0.193	0.286	0.255	0.327	0.211	0.302	0.193	**0.281**	0.228	0.319	**0.191**	0.286	0.226	0.312	0.236	0.313
	Avg.	0.159	0.254	0.158	0.250	0.217	0.295	0.168	0.261	0.158	**0.248**	0.180	0.277	**0.157**	0.253	0.175	0.267	0.188	0.273
Solar	96	0.170	0.204	0.175	0.228	0.209	0.224	0.218	0.235	**0.169**	**0.195**	0.239	0.285	0.202	0.263	0.192	0.207	0.682	0.688
	192	0.191	0.221	**0.186**	0.235	0.202	0.243	0.196	0.220	0.187	**0.207**	0.264	0.305	0.223	0.283	0.223	0.231	0.694	0.695
	336	0.201	0.231	0.200	0.246	0.251	0.250	**0.195**	0.220	0.199	**0.213**	0.289	0.325	0.241	0.292	0.243	0.250	0.719	0.709
	720	0.211	0.244	0.203	0.249	0.213	0.259	0.208	0.237	**0.202**	**0.216**	0.295	0.339	0.247	0.292	0.250	0.257	0.795	0.752
	Avg.	0.193	0.225	0.191	0.240	0.219	0.244	0.204	0.228	**0.189**	**0.208**	0.272	0.314	0.228	0.283	0.227	0.236	0.723	0.711
Traffic	96	**0.362**	0.250	0.366	0.259	0.651	0.391	0.384	0.250	0.365	**0.238**	0.429	0.313	0.368	0.253	0.365	0.240	0.383	0.257
	192	0.385	0.259	0.381	0.265	0.602	0.363	0.405	0.257	0.383	**0.249**	0.454	0.328	**0.379**	0.261	0.388	0.250	0.388	0.268
	336	**0.396**	0.271	0.397	0.269	0.609	0.366	0.424	0.265	0.397	0.259	0.474	0.341	0.397	0.270	0.397	**0.257**	0.424	0.270
	720	**0.435**	0.289	0.439	0.292	0.647	0.385	0.452	0.283	**0.435**	0.278	0.514	0.363	0.440	0.296	0.438	**0.271**	0.461	0.289
	Avg.	**0.395**	0.267	0.396	0.271	0.627	0.376	0.416	0.264	**0.395**	0.256	0.468	0.336	0.396	0.270	0.397	**0.255**	0.414	0.271

## Appendix E. Main Comparison: Run Variability

Table A5 and Table A6 report the mean and sample standard deviation (SD) across three runs for each dataset, horizon, method, and metric. The SD describes the variation in evaluation scores across runs. It is distinct from a confidence interval for the mean or a prediction interval for future observations. The mean results retain the precision of the corresponding comparison tables. SDs are shown to four decimal places. Horizon averages remain in the mean comparison tables; SDs are reported separately for each horizon.

**Table A5:** Main comparison results as mean ± SD across three runs. MSE and MAE occupy separate rows to keep all results readable.

Dataset	*H*	Metric	EFP	iTransformer	TimeMixer	PatchTST	Crossformer	TimesNet	DLinear
ETTh1	96	MSE	0.381±0.0073	0.386±0.0057	0.372±0.0031	0.377±0.0042	0.411±0.0104	0.389±0.0090	0.379±0.0031
		MAE	0.401±0.0017	0.405±0.0091	0.401±0.0070	0.397±0.0110	0.435±0.0087	0.412±0.0055	0.403±0.0051
ETTh1	192	MSE	0.405±0.0037	0.424±0.0035	0.413±0.0074	0.409±0.0036	0.409±0.0069	0.440±0.0042	0.408±0.0055
		MAE	0.429±0.0061	0.440±0.0021	0.430±0.0102	0.425±0.0035	0.438±0.0054	0.443±0.0037	0.419±0.0042
ETTh1	336	MSE	0.428±0.0034	0.449±0.0066	0.438±0.0055	0.431±0.0037	0.433±0.0054	0.523±0.0102	0.440±0.0062
		MAE	0.436±0.0098	0.460±0.0146	0.450±0.0028	0.444±0.0058	0.457±0.0113	0.487±0.0038	0.440±0.0076
ETTh1	720	MSE	0.451±0.0061	0.495±0.0119	0.486±0.0147	0.457±0.0145	0.501±0.0144	0.521±0.0036	0.471±0.0062
		MAE	0.472±0.0040	0.487±0.0131	0.484±0.0100	0.477±0.0076	0.514±0.0139	0.495±0.0033	0.493±0.0098
ETTh2	96	MSE	0.274±0.0010	0.297±0.0023	0.281±0.0060	0.277±0.0020	0.728±0.0184	0.334±0.0073	0.300±0.0043
		MAE	0.341±0.0034	0.348±0.0029	0.351±0.0034	0.337±0.0099	0.603±0.0124	0.370±0.0059	0.364±0.0066
ETTh2	192	MSE	0.346±0.0013	0.372±0.0090	0.349±0.0041	0.348±0.0056	0.723±0.0044	0.404±0.0075	0.387±0.0037
		MAE	0.387±0.0105	0.403±0.0087	0.389±0.0020	0.384±0.0054	0.607±0.0124	0.413±0.0063	0.423±0.0035
ETTh2	336	MSE	0.363±0.0088	0.388±0.0080	0.366±0.0036	0.377±0.0057	0.740±0.0212	0.389±0.0072	0.490±0.0143
		MAE	0.410±0.0039	0.417±0.0084	0.413±0.0053	0.416±0.0036	0.628±0.0091	0.435±0.0091	0.487±0.0117
ETTh2	720	MSE	0.396±0.0093	0.424±0.0039	0.401±0.0055	0.406±0.0058	1.386±0.0231	0.434±0.0061	0.704±0.0205
		MAE	0.432±0.0087	0.444±0.0100	0.436±0.0085	0.441±0.0092	0.882±0.0203	0.448±0.0094	0.597±0.0049
ETTm1	96	MSE	0.287±0.0043	0.300±0.0058	0.293±0.0082	0.289±0.0027	0.314±0.0065	0.340±0.0047	0.300±0.0064
		MAE	0.342±0.0060	0.353±0.0106	0.345±0.0101	0.343±0.0106	0.367±0.0061	0.378±0.0035	0.345±0.0065
ETTm1	192	MSE	0.325±0.0037	0.341±0.0077	0.335±0.0104	0.329±0.0078	0.374±0.0018	0.392±0.0040	0.336±0.0037
		MAE	0.366±0.0037	0.380±0.0039	0.372±0.0019	0.368±0.0098	0.410±0.0051	0.404±0.0091	0.369±0.0076
ETTm1	336	MSE	0.359±0.0064	0.374±0.0082	0.368±0.0048	0.362±0.0037	0.413±0.0041	0.423±0.0108	0.367±0.0091
		MAE	0.387±0.0029	0.396±0.0029	0.388±0.0051	0.390±0.0096	0.432±0.0046	0.426±0.0048	0.386±0.0090
ETTm1	720	MSE	0.412±0.0063	0.429±0.0057	0.426±0.0014	0.416±0.0018	0.753±0.0061	0.475±0.0078	0.419±0.0094
		MAE	0.410±0.0035	0.430±0.0071	0.417±0.0059	0.423±0.0049	0.613±0.0051	0.453±0.0050	0.416±0.0050
ETTm2	96	MSE	0.162±0.0049	0.175±0.0040	0.165±0.0017	0.165±0.0017	0.296±0.0059	0.189±0.0026	0.164±0.0015
		MAE	0.249±0.0065	0.266±0.0043	0.256±0.0032	0.255±0.0018	0.391±0.0083	0.265±0.0012	0.255±0.0035
ETTm2	192	MSE	0.219±0.0065	0.242±0.0050	0.225±0.0019	0.221±0.0026	0.369±0.0060	0.254±0.0079	0.224±0.0053
		MAE	0.291±0.0044	0.312±0.0045	0.298±0.0021	0.293±0.0076	0.416±0.0097	0.310±0.0044	0.304±0.0085
ETTm2	336	MSE	0.273±0.0042	0.282±0.0038	0.277±0.0040	0.276±0.0057	0.588±0.0128	0.313±0.0019	0.277±0.0012
		MAE	0.324±0.0070	0.337±0.0018	0.332±0.0033	0.327±0.0036	0.600±0.0122	0.345±0.0086	0.337±0.0063
ETTm2	720	MSE	0.356±0.0095	0.375±0.0098	0.360±0.0052	0.362±0.0025	0.750±0.0218	0.413±0.0059	0.371±0.0039
		MAE	0.378±0.0044	0.394±0.0019	0.387±0.0020	0.381±0.0031	0.612±0.0147	0.402±0.0109	0.401±0.0029
Exchange	96	MSE	0.080±0.0037	0.086±0.0024	0.084±0.0033	0.079±0.0041	0.088±0.0013	0.112±0.0033	0.080±0.0007
		MAE	0.200±0.0029	0.205±0.0038	0.207±0.0048	0.202±0.0032	0.213±0.0061	0.242±0.0077	0.202±0.0069
Exchange	192	MSE	0.161±0.0020	0.177±0.0049	0.178±0.0053	0.159±0.0064	0.157±0.0031	0.209±0.0024	0.182±0.0047
		MAE	0.287±0.0067	0.299±0.0042	0.300±0.0054	0.289±0.0074	0.288±0.0082	0.334±0.0073	0.321±0.0029
Exchange	336	MSE	0.296±0.0047	0.331±0.0082	0.376±0.0076	0.297±0.0065	0.332±0.0026	0.358±0.0049	0.327±0.0090
		MAE	0.396±0.0042	0.417±0.0033	0.451±0.0060	0.399±0.0031	0.429±0.0089	0.435±0.0083	0.434±0.0082
Exchange	720	MSE	0.581±0.0087	0.846±0.0112	0.884±0.0038	0.751±0.0181	0.980±0.0069	0.944±0.0097	0.583±0.0104
		MAE	0.603±0.0074	0.693±0.0156	0.707±0.0113	0.650±0.0166	0.762±0.0111	0.736±0.0183	0.605±0.0039
Weather	96	MSE	0.144±0.0021	0.157±0.0010	0.147±0.0062	0.149±0.0014	0.143±0.0050	0.168±0.0028	0.170±0.0064
		MAE	0.198±0.0064	0.207±0.0083	0.201±0.0024	0.196±0.0049	0.210±0.0019	0.214±0.0025	0.230±0.0062
Weather	192	MSE	0.190±0.0026	0.200±0.0059	0.192±0.0044	0.191±0.0049	0.198±0.0030	0.219±0.0032	0.216±0.0076
		MAE	0.237±0.0053	0.248±0.0032	0.243±0.0025	0.239±0.0012	0.260±0.0082	0.262±0.0026	0.273±0.0027
Weather	336	MSE	0.239±0.0034	0.252±0.0010	0.247±0.0011	0.242±0.0016	0.258±0.0069	0.278±0.0027	0.258±0.0062
		MAE	0.276±0.0083	0.287±0.0081	0.284±0.0040	0.279±0.0046	0.314±0.0062	0.302±0.0014	0.307±0.0059
Weather	720	MSE	0.311±0.0067	0.320±0.0065	0.318±0.0063	0.313±0.0095	0.335±0.0021	0.353±0.0041	0.323±0.0077
		MAE	0.322±0.0016	0.336±0.0020	0.330±0.0075	0.330±0.0110	0.385±0.0063	0.351±0.0053	0.362±0.0094
Electricity	96	MSE	0.130±0.0033	0.134±0.0022	0.153±0.0033	0.143±0.0058	0.134±0.0033	0.169±0.0026	0.140±0.0021
		MAE	0.225±0.0060	0.230±0.0013	0.256±0.0041	0.247±0.0047	0.231±0.0055	0.271±0.0061	0.237±0.0013
Electricity	192	MSE	0.146±0.0011	0.154±0.0040	0.168±0.0008	0.158±0.0040	0.147±0.0042	0.180±0.0025	0.154±0.0023
		MAE	0.241±0.0025	0.250±0.0022	0.269±0.0046	0.260±0.0038	0.243±0.0032	0.280±0.0049	0.251±0.0012
Electricity	336	MSE	0.165±0.0046	0.169±0.0034	0.189±0.0048	0.168±0.0054	0.165±0.0030	0.204±0.0044	0.169±0.0032
		MAE	0.262±0.0022	0.265±0.0041	0.291±0.0018	0.267±0.0016	0.264±0.0030	0.304±0.0101	0.268±0.0039
Electricity	720	MSE	0.195±0.0041	0.197±0.0014	0.228±0.0057	0.214±0.0031	0.237±0.0059	0.205±0.0047	0.204±0.0032
		MAE	0.289±0.0010	0.292±0.0018	0.320±0.0092	0.307±0.0083	0.314±0.0044	0.304±0.0056	0.301±0.0030
Solar	96	MSE	0.170±0.0012	0.190±0.0015	0.179±0.0064	0.172±0.0058	0.183±0.0020	0.198±0.0059	0.199±0.0048
		MAE	0.204±0.0030	0.244±0.0014	0.232±0.0012	0.234±0.0047	0.208±0.0024	0.270±0.0052	0.265±0.0019
Solar	192	MSE	0.191±0.0062	0.193±0.0050	0.201±0.0027	0.204±0.0083	0.208±0.0033	0.206±0.0056	0.220±0.0009
		MAE	0.221±0.0066	0.257±0.0060	0.259±0.0037	0.302±0.0041	0.226±0.0075	0.276±0.0076	0.282±0.0013
Solar	336	MSE	0.201±0.0056	0.203±0.0016	0.190±0.0058	0.212±0.0031	0.212±0.0045	0.208±0.0079	0.234±0.0011
		MAE	0.231±0.0062	0.266±0.0064	0.256±0.0042	0.293±0.0022	0.239±0.0068	0.284±0.0024	0.295±0.0010
Solar	720	MSE	0.211±0.0051	0.223±0.0019	0.203±0.0047	0.215±0.0011	0.215±0.0017	0.232±0.0016	0.243±0.0030
		MAE	0.244±0.0059	0.281±0.0083	0.261±0.0022	0.307±0.0088	0.256±0.0017	0.294±0.0083	0.301±0.0107
Traffic	96	MSE	0.362±0.0084	0.364±0.0054	0.369±0.0070	0.370±0.0056	0.526±0.0100	0.595±0.0090	0.395±0.0016
		MAE	0.250±0.0045	0.265±0.0071	0.257±0.0067	0.262±0.0017	0.288±0.0038	0.312±0.0034	0.275±0.0013
Traffic	192	MSE	0.385±0.0021	0.384±0.0086	0.400±0.0082	0.386±0.0028	0.503±0.0144	0.613±0.0096	0.407±0.0032
		MAE	0.259±0.0049	0.273±0.0034	0.272±0.0015	0.269±0.0043	0.263±0.0024	0.322±0.0016	0.280±0.0025
Traffic	336	MSE	0.396±0.0020	0.398±0.0054	0.407±0.0021	0.396±0.0078	0.505±0.0032	0.626±0.0104	0.417±0.0057
		MAE	0.271±0.0042	0.277±0.0022	0.272±0.0035	0.275±0.0046	0.276±0.0077	0.332±0.0087	0.286±0.0020
Traffic	720	MSE	0.435±0.0046	0.445±0.0024	0.461±0.0070	0.435±0.0031	0.552±0.0064	0.635±0.0088	0.454±0.0076
		MAE	0.289±0.0062	0.308±0.0043	0.316±0.0065	0.295±0.0041	0.301±0.0053	0.340±0.0029	0.308±0.0078

## Appendix F. Additional Comparison: Run Variability

**Table A6:** Additional baseline results as mean ± SD across three runs. The EFP entries are repeated for comparison.

Dataset	*H*	Metric	EFP	AMD	FITS	Pathformer	DUET	SimpleTM	ModernTCN	TimeBridge	MOIRAI
ETTh1	96	MSE	0.381±0.0073	0.369±0.0038	0.386±0.0051	0.372±0.0063	0.352±0.0024	0.390±0.0020	0.368±0.0102	0.375±0.0087	0.380±0.0078
		MAE	0.401±0.0017	0.397±0.0075	0.396±0.0048	0.392±0.0029	0.384±0.0049	0.410±0.0042	0.394±0.0086	0.390±0.0033	0.398±0.0040
ETTh1	192	MSE	0.405±0.0037	0.407±0.0092	0.436±0.0012	0.408±0.0079	0.398±0.0050	0.422±0.0013	0.405±0.0054	0.428±0.0089	0.440±0.0051
		MAE	0.429±0.0061	0.416±0.0090	0.423±0.0032	0.415±0.0045	0.409±0.0029	0.428±0.0025	0.413±0.0074	0.421±0.0072	0.434±0.0059
ETTh1	336	MSE	0.428±0.0034	0.418±0.0023	0.478±0.0075	0.438±0.0062	0.414±0.0072	0.440±0.0096	0.391±0.0097	0.470±0.0102	0.514±0.0059
		MAE	0.436±0.0098	0.427±0.0016	0.444±0.0098	0.434±0.0040	0.426±0.0090	0.440±0.0057	0.412±0.0046	0.443±0.0083	0.474±0.0139
ETTh1	720	MSE	0.451±0.0061	0.439±0.0076	0.502±0.0071	0.450±0.0019	0.429±0.0088	0.459±0.0030	0.450±0.0126	0.481±0.0056	0.705±0.0216
		MAE	0.472±0.0040	0.454±0.0138	0.495±0.0069	0.463±0.0137	0.455±0.0031	0.475±0.0078	0.461±0.0033	0.481±0.0103	0.568±0.0062
ETTh2	96	MSE	0.274±0.0010	0.274±0.0083	0.295±0.0074	0.279±0.0062	0.276±0.0074	0.298±0.0052	0.263±0.0044	0.286±0.0021	0.287±0.0040
		MAE	0.341±0.0034	0.337±0.0047	0.350±0.0071	0.336±0.0037	0.336±0.0089	0.349±0.0082	0.332±0.0101	0.333±0.0085	0.325±0.0064
ETTh2	192	MSE	0.346±0.0013	0.351±0.0105	0.381±0.0108	0.345±0.0097	0.332±0.0106	0.379±0.0077	0.320±0.0085	0.364±0.0063	0.347±0.0016
		MAE	0.387±0.0105	0.383±0.0025	0.396±0.0100	0.380±0.0038	0.374±0.0099	0.396±0.0083	0.374±0.0043	0.385±0.0024	0.367±0.0080
ETTh2	336	MSE	0.363±0.0088	0.375±0.0048	0.426±0.0021	0.378±0.0080	0.353±0.0029	0.400±0.0022	0.313±0.0087	0.416±0.0098	0.377±0.0079
		MAE	0.410±0.0039	0.411±0.0076	0.438±0.0083	0.408±0.0045	0.397±0.0028	0.420±0.0080	0.376±0.0110	0.425±0.0087	0.393±0.0067
ETTh2	720	MSE	0.396±0.0093	0.402±0.0037	0.431±0.0030	0.437±0.0013	0.382±0.0099	0.432±0.0054	0.397±0.0098	0.435±0.0014	0.404±0.0086
		MAE	0.432±0.0087	0.438±0.0079	0.446±0.0069	0.455±0.0038	0.425±0.0071	0.451±0.0038	0.433±0.0057	0.448±0.0068	0.421±0.0101
ETTm1	96	MSE	0.287±0.0043	0.284±0.0041	0.355±0.0069	0.290±0.0061	0.279±0.0018	0.286±0.0032	0.292±0.0021	0.312±0.0107	0.353±0.0025
		MAE	0.342±0.0060	0.339±0.0022	0.375±0.0017	0.335±0.0031	0.333±0.0022	0.338±0.0044	0.346±0.0073	0.344±0.0046	0.363±0.0089
ETTm1	192	MSE	0.325±0.0037	0.328±0.0085	0.392±0.0078	0.337±0.0028	0.320±0.0063	0.326±0.0100	0.332±0.0076	0.366±0.0060	0.376±0.0076
		MAE	0.366±0.0037	0.362±0.0071	0.393±0.0049	0.363±0.0066	0.358±0.0055	0.364±0.0041	0.368±0.0035	0.377±0.0058	0.380±0.0035
ETTm1	336	MSE	0.359±0.0064	0.360±0.0060	0.424±0.0058	0.374±0.0074	0.348±0.0088	0.364±0.0044	0.365±0.0034	0.403±0.0105	0.399±0.0085
		MAE	0.387±0.0029	0.380±0.0023	0.414±0.0093	0.384±0.0097	0.377±0.0029	0.388±0.0101	0.391±0.0061	0.404±0.0025	0.395±0.0021
ETTm1	720	MSE	0.412±0.0063	0.421±0.0055	0.487±0.0124	0.428±0.0098	0.405±0.0020	0.424±0.0109	0.416±0.0064	0.454±0.0076	0.432±0.0080
		MAE	0.410±0.0035	0.416±0.0109	0.449±0.0068	0.416±0.0082	0.408±0.0098	0.426±0.0092	0.417±0.0058	0.438±0.0040	0.417±0.0095
ETTm2	96	MSE	0.162±0.0049	0.167±0.0048	0.183±0.0062	0.164±0.0052	0.161±0.0051	0.174±0.0038	0.166±0.0063	0.175±0.0043	0.189±0.0063
		MAE	0.249±0.0065	0.258±0.0069	0.266±0.0065	0.250±0.0017	0.248±0.0063	0.257±0.0064	0.256±0.0072	0.252±0.0041	0.260±0.0059
ETTm2	192	MSE	0.219±0.0065	0.221±0.0078	0.247±0.0064	0.219±0.0063	0.214±0.0018	0.233±0.0017	0.222±0.0011	0.246±0.0019	0.247±0.0051
		MAE	0.291±0.0044	0.294±0.0074	0.305±0.0068	0.288±0.0039	0.286±0.0068	0.297±0.0058	0.293±0.0079	0.299±0.0037	0.300±0.0022
ETTm2	336	MSE	0.273±0.0042	0.270±0.0012	0.307±0.0012	0.267±0.0023	0.267±0.0052	0.279±0.0082	0.272±0.0084	0.311±0.0104	0.295±0.0035
		MAE	0.324±0.0070	0.327±0.0060	0.342±0.0075	0.319±0.0088	0.321±0.0105	0.330±0.0031	0.324±0.0029	0.341±0.0061	0.334±0.0024
ETTm2	720	MSE	0.356±0.0095	0.356±0.0109	0.407±0.0107	0.361±0.0093	0.348±0.0090	0.367±0.0059	0.358±0.0031	0.403±0.0096	0.372±0.0044
		MAE	0.378±0.0044	0.382±0.0099	0.399±0.0039	0.377±0.0064	0.374±0.0082	0.387±0.0037	0.381±0.0101	0.397±0.0050	0.386±0.0076
Exchange	96	MSE	0.080±0.0037	0.083±0.0028	0.089±0.0026	0.088±0.0031	0.080±0.0024	0.093±0.0029	0.095±0.0015	0.091±0.0029	0.090±0.0030
		MAE	0.200±0.0029	0.201±0.0037	0.202±0.0018	0.208±0.0037	0.198±0.0011	0.211±0.0080	0.206±0.0037	0.214±0.0037	0.204±0.0060
Exchange	192	MSE	0.161±0.0020	0.171±0.0025	0.165±0.0032	0.183±0.0053	0.162±0.0051	0.166±0.0015	0.170±0.0030	0.164±0.0056	0.168±0.0017
		MAE	0.287±0.0067	0.293±0.0061	0.293±0.0011	0.304±0.0030	0.288±0.0022	0.288±0.0023	0.291±0.0031	0.295±0.0053	0.295±0.0060
Exchange	336	MSE	0.296±0.0047	0.309±0.0086	0.298±0.0075	0.354±0.0091	0.294±0.0048	0.308±0.0033	0.319±0.0066	0.306±0.0034	0.302±0.0069
		MAE	0.396±0.0042	0.402±0.0047	0.397±0.0059	0.429±0.0024	0.392±0.0058	0.408±0.0090	0.402±0.0081	0.400±0.0048	0.405±0.0029
Exchange	720	MSE	0.581±0.0087	0.750±0.0049	0.778±0.0199	0.909±0.0089	0.583±0.0043	0.767±0.0143	0.765±0.0098	0.770±0.0171	0.620±0.0105
		MAE	0.603±0.0074	0.652±0.0147	0.624±0.0077	0.716±0.0032	0.580±0.0096	0.652±0.0110	0.604±0.0074	0.631±0.0067	0.648±0.0046
Weather	96	MSE	0.144±0.0021	0.145±0.0049	0.166±0.0063	0.148±0.0023	0.146±0.0022	0.148±0.0050	0.149±0.0037	0.169±0.0055	0.177±0.0010
		MAE	0.198±0.0064	0.197±0.0036	0.213±0.0009	0.195±0.0048	0.191±0.0029	0.197±0.0064	0.200±0.0028	0.206±0.0014	0.208±0.0038
Weather	192	MSE	0.190±0.0026	0.187±0.0008	0.213±0.0080	0.191±0.0007	0.190±0.0050	0.195±0.0031	0.196±0.0033	0.219±0.0038	0.219±0.0010
		MAE	0.237±0.0053	0.238±0.0059	0.254±0.0079	0.235±0.0045	0.231±0.0020	0.244±0.0081	0.245±0.0027	0.249±0.0073	0.249±0.0063
Weather	336	MSE	0.239±0.0034	0.240±0.0074	0.269±0.0065	0.243±0.0060	0.234±0.0072	0.249±0.0067	0.241±0.0081	0.274±0.0033	0.277±0.0021
		MAE	0.276±0.0083	0.280±0.0047	0.294±0.0066	0.274±0.0044	0.268±0.0034	0.287±0.0028	0.277±0.0046	0.290±0.0040	0.292±0.0046
Weather	720	MSE	0.311±0.0067	0.315±0.0108	0.346±0.0109	0.318±0.0039	0.305±0.0028	0.329±0.0021	0.314±0.0087	0.353±0.0099	0.365±0.0036
		MAE	0.322±0.0016	0.330±0.0108	0.343±0.0017	0.326±0.0031	0.319±0.0091	0.339±0.0027	0.334±0.0029	0.341±0.0093	0.350±0.0039
Electricity	96	MSE	0.130±0.0033	0.130±0.0042	0.200±0.0072	0.135±0.0059	0.128±0.0035	0.146±0.0029	0.131±0.0014	0.138±0.0024	0.152±0.0034
		MAE	0.225±0.0060	0.224±0.0079	0.278±0.0074	0.222±0.0033	0.219±0.0047	0.245±0.0013	0.226±0.0070	0.232±0.0041	0.242±0.0050
Electricity	192	MSE	0.146±0.0011	0.147±0.0037	0.200±0.0057	0.157±0.0017	0.148±0.0059	0.164±0.0028	0.143±0.0058	0.157±0.0049	0.171±0.0044
		MAE	0.241±0.0025	0.238±0.0066	0.280±0.0065	0.253±0.0047	0.235±0.0029	0.262±0.0031	0.239±0.0084	0.251±0.0048	0.259±0.0078
Electricity	336	MSE	0.165±0.0046	0.160±0.0038	0.214±0.0025	0.170±0.0023	0.163±0.0065	0.183±0.0057	0.161±0.0019	0.178±0.0025	0.192±0.0043
		MAE	0.262±0.0022	0.253±0.0016	0.295±0.0024	0.267±0.0081	0.255±0.0066	0.281±0.0078	0.259±0.0017	0.272±0.0027	0.278±0.0078
Electricity	720	MSE	0.195±0.0041	0.193±0.0048	0.255±0.0039	0.211±0.0057	0.193±0.0018	0.228±0.0030	0.191±0.0025	0.226±0.0020	0.236±0.0018
		MAE	0.289±0.0010	0.286±0.0063	0.327±0.0066	0.302±0.0063	0.281±0.0055	0.319±0.0108	0.286±0.0080	0.312±0.0016	0.313±0.0098
Solar	96	MSE	0.170±0.0012	0.175±0.0042	0.209±0.0015	0.218±0.0070	0.169±0.0008	0.239±0.0065	0.202±0.0032	0.192±0.0034	0.682±0.0183
		MAE	0.204±0.0030	0.228±0.0069	0.224±0.0080	0.235±0.0052	0.195±0.0022	0.285±0.0043	0.263±0.0027	0.207±0.0040	0.688±0.0098
Solar	192	MSE	0.191±0.0062	0.186±0.0008	0.202±0.0078	0.196±0.0054	0.187±0.0032	0.264±0.0038	0.223±0.0023	0.223±0.0022	0.694±0.0202
		MAE	0.221±0.0066	0.235±0.0050	0.243±0.0041	0.220±0.0040	0.207±0.0041	0.305±0.0091	0.283±0.0051	0.231±0.0030	0.695±0.0081
Solar	336	MSE	0.201±0.0056	0.200±0.0076	0.251±0.0081	0.195±0.0051	0.199±0.0026	0.289±0.0017	0.241±0.0045	0.243±0.0061	0.719±0.0061
		MAE	0.231±0.0062	0.246±0.0028	0.250±0.0082	0.220±0.0013	0.213±0.0023	0.325±0.0068	0.292±0.0014	0.250±0.0043	0.709±0.0155
Solar	720	MSE	0.211±0.0051	0.203±0.0014	0.213±0.0066	0.208±0.0047	0.202±0.0038	0.295±0.0063	0.247±0.0072	0.250±0.0031	0.795±0.0193
		MAE	0.244±0.0059	0.249±0.0060	0.259±0.0015	0.237±0.0062	0.216±0.0072	0.339±0.0030	0.292±0.0030	0.257±0.0028	0.752±0.0113
Traffic	96	MSE	0.362±0.0084	0.366±0.0094	0.651±0.0152	0.384±0.0055	0.365±0.0049	0.429±0.0059	0.368±0.0070	0.365±0.0070	0.383±0.0049
		MAE	0.250±0.0045	0.259±0.0017	0.391±0.0085	0.250±0.0064	0.238±0.0081	0.313±0.0081	0.253±0.0033	0.240±0.0064	0.257±0.0022
Traffic	192	MSE	0.385±0.0021	0.381±0.0041	0.602±0.0068	0.405±0.0052	0.383±0.0065	0.454±0.0117	0.379±0.0110	0.388±0.0101	0.388±0.0092
		MAE	0.259±0.0049	0.265±0.0075	0.363±0.0063	0.257±0.0037	0.249±0.0075	0.328±0.0037	0.261±0.0036	0.250±0.0055	0.268±0.0075
Traffic	336	MSE	0.396±0.0020	0.397±0.0044	0.609±0.0067	0.424±0.0069	0.397±0.0014	0.474±0.0113	0.397±0.0076	0.397±0.0068	0.424±0.0068
		MAE	0.271±0.0042	0.269±0.0063	0.366±0.0104	0.265±0.0018	0.259±0.0049	0.341±0.0039	0.270±0.0041	0.257±0.0015	0.270±0.0055
Traffic	720	MSE	0.435±0.0046	0.439±0.0070	0.647±0.0059	0.452±0.0065	0.435±0.0099	0.514±0.0072	0.440±0.0068	0.438±0.0020	0.461±0.0023
		MAE	0.289±0.0062	0.292±0.0041	0.385±0.0044	0.283±0.0050	0.278±0.0052	0.363±0.0074	0.296±0.0067	0.271±0.0010	0.289±0.0036

## Appendix G. Statistical Comparisons

We compare EFP with all 14 baselines in the main and additional comparisons and with the Value Forecast control. The MSE and MAE are tested separately. For each method, we first average the reported means over the four horizons within each dataset. We then average ETTh1, ETTh2, ETTm1, and ETTm2 into one ETT group because they are related measurements from the same benchmark family. The remaining groups are Exchange, Weather, Electricity, Solar, and Traffic. Each of these six groups contributes one paired difference. This gives ETT the same weight as each other group and keeps related horizons out of the sample count. For Value Forecast, we use the dataset averages reported in its control table, including its corresponding EFP values at four decimal places.

We use the Wilcoxon signed rank test [43] with a two-sided alternative. The null model assumes independent group differences with distributions symmetric about zero. We discard zero differences, assign average ranks to tied absolute differences, and enumerate every sign assignment to obtain exact *p* values. Holm correction [44] is applied jointly to all 30 comparisons at a family significance level of 0.05. This analysis tests differences across benchmark groups; the SD tables describe the separate source of variation across repeated runs.

**Table A7:** Paired comparisons across six benchmark groups. *D* is the mean group difference (comparator minus EFP); positive values favor EFP. *p* is the exact unadjusted value, and $p_{H}$ is the Holm-adjusted value across all 30 tests.

Comparator	MSE			MAE		
	D	p	$p_{H}$	D	p	$p_{H}$
iTransformer	0.0214	0.0312	0.9375	0.0187	0.0312	0.9375
TimeMixer	0.0256	0.0625	0.9375	0.0209	0.0312	0.9375
PatchTST	0.0117	0.0312	0.9375	0.0170	0.0312	0.9375
Crossformer	0.0844	0.0312	0.9375	0.0439	0.0312	0.9375
TimesNet	0.0801	0.0312	0.9375	0.0445	0.0312	0.9375
DLinear	0.0223	0.0312	0.9375	0.0281	0.0312	0.9375
AMD	0.0079	0.8438	1.0000	0.0051	0.2500	1.0000
FITS	0.0735	0.0312	0.9375	0.0352	0.0312	0.9375
Pathformer	0.0261	0.0312	0.9375	0.0076	0.5625	1.0000
DUET	−0.0027	0.1562	1.0000	−0.0095	0.0312	0.9375
SimpleTM	0.0418	0.0312	0.9375	0.0355	0.0312	0.9375
ModernTCN	0.0148	0.4375	1.0000	0.0105	0.3125	1.0000
TimeBridge	0.0281	0.0312	0.9375	0.0076	0.2188	1.0000
MOIRAI	0.1123	0.0312	0.9375	0.0919	0.0312	0.9375
Value Forecast	0.0123	0.0625	0.9375	0.0113	0.1562	1.0000

No comparison reaches the 0.05 threshold after correction. With six paired groups, the exact *p* values have a coarse resolution: the smallest possible two-sided value is $2/2^{6}=0.03125$. The error differences therefore describe the observed benchmark performance, while the corrected tests do not establish a significant difference for any individual comparator. This result does not establish equivalence between methods.
